# Endothelial-specific Ezh2 deficiency exacerbates blood-brain barrier dysfunction and neuroinflammation in sepsis-associated encephalopathy

**DOI:** 10.1186/s12974-026-03798-z

**Published:** 2026-04-07

**Authors:** Hui Zhu, Qiuwen Sun, Ying Wang, Xiangyu Hu, Yulin Pan, Tianyi Huang, Siyuan Xu, Li Mu, Yiting Wang, Muyuan Tong, Yuqian Tang, Zhiwen Luo, Bucheng Yang, Minghao Hou, Yanzi Chen, Yiting Chen, Xing Su, Xi Xu, Zhiping Wang, Maohong Cao, Chengbin Xue

**Affiliations:** 1 Research Center of Clinical Medicine, Clinical and Translational Research Center, Jiangsu Key Laboratory of Tissue Engineering and Neuroregeneration, Key Laboratory of Neuroregeneration of Ministry of Education, Co-innovation Center of Neuroregeneration, NMPA Key Laboratory for Research and Evaluation of Tissue Engineering Technology Products, Affiliated Hospital of Nantong University, Nantong University, Nantong, 226001 China; 2 Medical School of Nantong University, Nantong, JS 226001 P.R. China; 3 Department of Neurology, Affiliated Hospital of Nantong University, Nantong, JS 226001 P. R. China; 4 Department of Neurosurgery, Affiliated Hospital of Nantong University, Nantong, 226001 China; 5 Department of Rehabilitation Medicine, Affiliated Hospital of Nantong University, Nantong, JS 226001 P. R. China; 6 Department of Critical Care Medicine, Nantong Fourth People’s Hospital, Nantong, JS 226001 P. R. China

**Keywords:** *Ezh2*, Endothelial cells, Sepsis, Sepsis-associated encephalopathy, Neuroinflammation

## Abstract

Sepsis-associated encephalopathy (SAE) is a frequent yet insufficiently understood form of acute brain dysfunction driven by systemic inflammation and neurovascular injury. Here, we identify endothelial Enhancer of Zeste Homolog 2 (*Ezh2*) as a key epigenetic regulator that maintains blood-brain barrier (BBB) integrity and restrains neuroinflammatory responses during sepsis. Using constitutive and inducible endothelial-specific *Ezh2* knockout mice, we demonstrate that loss of *Ezh2* disrupts neurovascular unit organization at baseline, characterized by reduced tight junction protein expression, diminished glucose transporter levels, altered vascular morphology, and impaired astrocytic endfoot coverage. Following cecal ligation and puncture, *Ezh2*-deficient mice exhibited markedly increased mortality, exacerbated cerebral edema, and severe albumin extravasation, indicating profound BBB breakdown. Neuropathological analyses revealed region-dependent neuronal degeneration and apoptosis across cortex, midbrain, and hippocampus, accompanied by reactive gliosis and microglial activation. The absence of *Ezh2* further promoted robust leukocyte infiltration, including neutrophils and CD4⁺/CD8⁺ T cells, into the cortex, basal ganglia, and choroid plexus, consistent with dysregulated peripheral-central nervous system (CNS) immune communication. Transcriptomic profiling identified upregulation of inflammatory, cytokine, and cell death pathways alongside downregulation of synaptic, metabolic, and neuronal maturation programs, providing a mechanistic molecular landscape linking endothelial epigenetic dysfunction to CNS vulnerability. Collectively, these findings reveal that endothelial *Ezh2* acts as a protective neurovascular gatekeeper that limits systemic inflammatory penetration into the CNS and mitigates neuroimmune activation during sepsis. This work establishes an epigenetic axis coupling BBB dysfunction to neuroinflammation in SAE and suggests that endothelial-targeted epigenetic modulation may represent a therapeutic strategy for limiting CNS injury in systemic inflammatory disorders.

**Graphical Abstract:**

**Figure:**
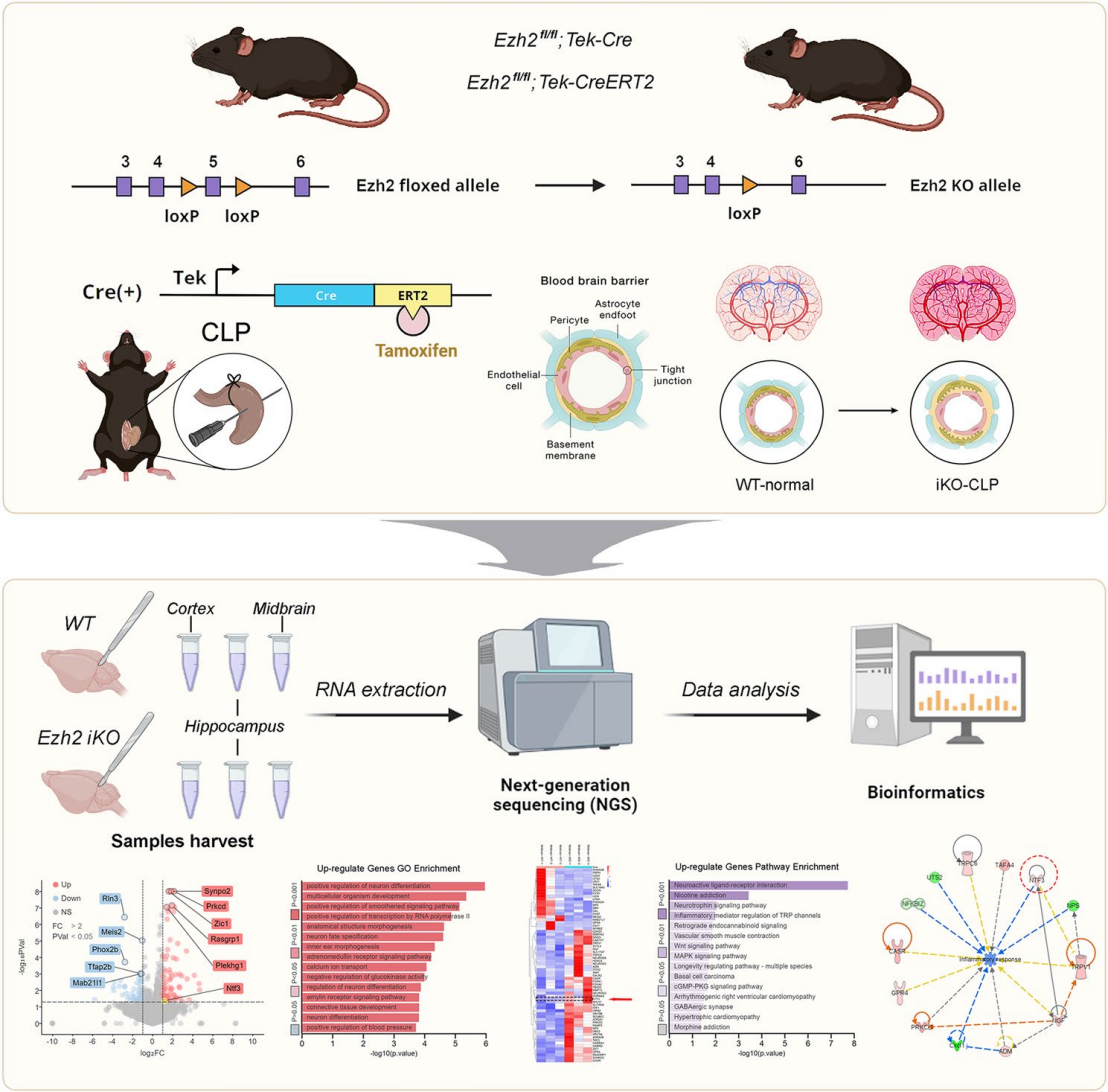
Endothelial *Ezh2* Regulates Neuroinflammation and Neuronal Apoptosis in Sepsis. This Graphic Abstract illustrates the experimental workflow and key findings regarding the role of endothelial-specific *Ezh2* in the brain during sepsis. The study utilizes two genetic mouse models: *Ezh2*^fl/fl^; Tek-Cre for conditional endothelial *Ezh2* knockout (*Ezh2* cKO) and *Ezh2*^fl/fl^; Tek-CreERT2 for tamoxifen-inducible endothelial *Ezh2* knockout (*Ezh2* iKO). These models allow for the specific deletion of *Ezh2* in endothelial cells, which form the crucial BBB. Sepsis is induced via the Cecal Ligation and Puncture (CLP) procedure. Following CLP, various brain regions, including the cortex, midbrain, and hippocampus, are harvested for RNA extraction and Next-Generation Sequencing (NGS) to identify differentially expressed genes. Subsequent bioinformatics analysis, including volcano plots, Gene Ontology (GO) enrichment, KEGG pathway analysis, heatmaps, and protein-protein interaction (PPI) networks, reveals significant alterations in gene expression related to inflammation, neuronal death, and BBB integrity in *Ezh2*-deficient mice compared to controls. The findings indicate that endothelial *Ezh2* plays a critical protective role in maintaining BBB function, mitigating neuroinflammation, and preventing neuronal apoptosis during sepsis

**Supplementary Information:**

The online version contains supplementary material available at 10.1186/s12974-026-03798-z.

## Introduction

Sepsis, a life-threatening condition characterized by systemic inflammation and multi-organ dysfunction, remains a significant global health challenge with high morbidity and mortality [1]. Central to the pathogenesis of sepsis is endothelial dysfunction, which leads to vascular leakage, impaired microcirculation, and organ failure [2]. While extensive research has advanced our understanding of the molecular mechanisms underlying sepsis [3, 4], the role of specific epigenetic regulators [5], particularly in vascular endothelial cells (ECs), remains poorly defined.

Sepsis-associated encephalopathy (SAE) is a complex, multifactorial syndrome characterized by acute brain dysfunction in the context of systemic infection without overt central nervous system (CNS) infection [6–8]. It is a frequent yet underdiagnosed complication of sepsis and is associated with significant morbidity and mortality [9]. Despite its prevalence, the pathophysiology of SAE remains incompletely understood, posing challenges for effective diagnosis and management [10].

The clinical manifestations of SAE are diverse, ranging from mild confusion and delirium to deep coma [11]. These symptoms often correlate poorly with the severity of systemic infection, underscoring the intricate interplay between systemic inflammatory processes and CNS dysfunction. Current evidence suggests that the underlying mechanisms of SAE involve a combination of neuroinflammation, BBB dysfunction, oxidative stress, mitochondrial dysfunction, and altered neurotransmission [12]. Each of these processes contributes to neuronal injury and impaired cerebral autoregulation, which further exacerbate neurological deficits [13].

Emerging research highlights the pivotal role of systemic inflammatory mediators, such as cytokines and chemokines, in disrupting the BBB and promoting neuroinflammation [14, 15]. Additionally, microglial activation and astrocytic dysfunction appear to play key roles in the progression of neuronal damage [16, 17]. Recent advances in neuroimaging and biomarker discovery have begun to elucidate the molecular and cellular pathways implicated in SAE, providing potential avenues for early diagnosis and therapeutic intervention [18].

From a clinical perspective, SAE represents a diagnostic challenge due to its nonspecific presentation and the absence of definitive diagnostic criteria [11]. The condition is often identified through exclusion, requiring a comprehensive evaluation to rule out alternative causes of encephalopathy [19]. Tools such as electroencephalography, magnetic resonance imaging, and cerebrospinal fluid analysis may aid in the assessment of SAE, though their utility is limited by variability in findings [20]. Therapeutic strategies for SAE remain largely supportive, emphasizing the management of the underlying sepsis and optimization of systemic hemodynamics and oxygenation [21]. However, targeted therapies addressing specific pathophysiological pathways are currently lacking. Preclinical studies exploring anti-inflammatory agents, antioxidants, and BBB stabilizers hold promise but have yet to translate into clinical practice.

Given its significant impact on patient outcomes, SAE has garnered increasing attention in critical care and neuroscience research [22]. Future investigations aimed at unraveling the complex mechanisms of SAE, refining diagnostic criteria, and developing targeted therapies are imperative. This article seeks to provide a comprehensive review of the current understanding of SAE, encompassing its pathophysiology, clinical features, diagnostic approaches, and therapeutic options, while highlighting key areas for future research.

The enhancer of zeste homolog 2 (*Ezh2*) gene encodes the catalytic subunit of polycomb repressive complex 2 (PRC2), which mediates histone H3 lysine 27 trimethylation (H3K27me3)-a key epigenetic mark associated with gene silencing [23]. *Ezh2* has been widely studied in contexts such as embryogenesis [24], cancer [25], and immune regulation [26], where it governs cell differentiation, proliferation, and inflammatory responses [27]. However, its role in maintaining vascular integrity and modulating inflammatory processes in endothelial cells has not been thoroughly explored. Notably, global deletion of *Ezh2* often results in embryonic lethality [28], further complicating investigations into its tissue-specific functions.

This study addresses this gap by presenting the first evidence that endothelial-specific knockout of *Ezh2* in mice, achieved using an inducible Cre-loxP system, does not cause embryonic lethality but compromises vascular integrity to a certain extent, contrary to a previous work from another group [29]. Importantly, in a mouse model of sepsis, endothelial-specific *Ezh2* deletion led to a significant increase in mortality, aggravated inflammatory responses in brain regions such as the cortex, hippocampus, and midbrain, and exacerbated reactivity of microglia and astrocytes in the present work. These observations highlight the critical role of endothelial *Ezh2* in orchestrating vascular and neuroimmune responses during sepsis.

To elucidate the underlying mechanisms, we performed transcriptomic analyses of brain regions to compare the effects of endothelial-specific *Ezh2* deletion on neurons, microglia, and astrocytes. These analyses revealed significant alterations in pathways related to microglial and astrocytic activation, neuronal survival, and vascular function. These molecular insights underscore the multifaceted role of *Ezh2* in endothelial cells in modulating neuroinflammation and preserving vascular homeostasis during sepsis.

The present work uncovers a novel function of endothelial *Ezh2* in mitigating sepsis progression by integrating vascular and neuroimmune mechanisms. Compared to previous studies focusing on *Ezh2*’s role in other cellular contexts [30, 31], this work uniquely demonstrates its endothelial-specific contributions to sepsis outcomes, offering new perspectives for therapeutic interventions. These findings pave the way for developing targeted therapeutic strategies aimed at modulating endothelial *Ezh2* activity to mitigate the severity of sepsis and its associated complications. Such interventions hold significant potential to improve clinical outcomes in patients suffering from this devastating condition.

## Materials and methods

### Animals

All animal procedures were performed in accordance with the Guide for the Care and Use of Laboratory Animals of the National Institutes of Health and approved by the Institutional Animal Care and Use Committee (IACUC) of Nantong University. The *Ezh2* flox, Tek-Cre and Tek-CreERT2 mice were purchased from the Shanghai Model Organisms Center, Inc. (Shanghai, China). The B6-G/R mice were purchased from GemPharmatech Co. Ltd (Nanjing, China). All experimental protocols were approved by the Administration Committee of Experimental Animals, Jiangsu Province, China, in accordance with the guidelines of the Institutional Animal Care and Use Committee, Nantong University, China (Inspection No: 20190225-004).

**Fig. 1:**
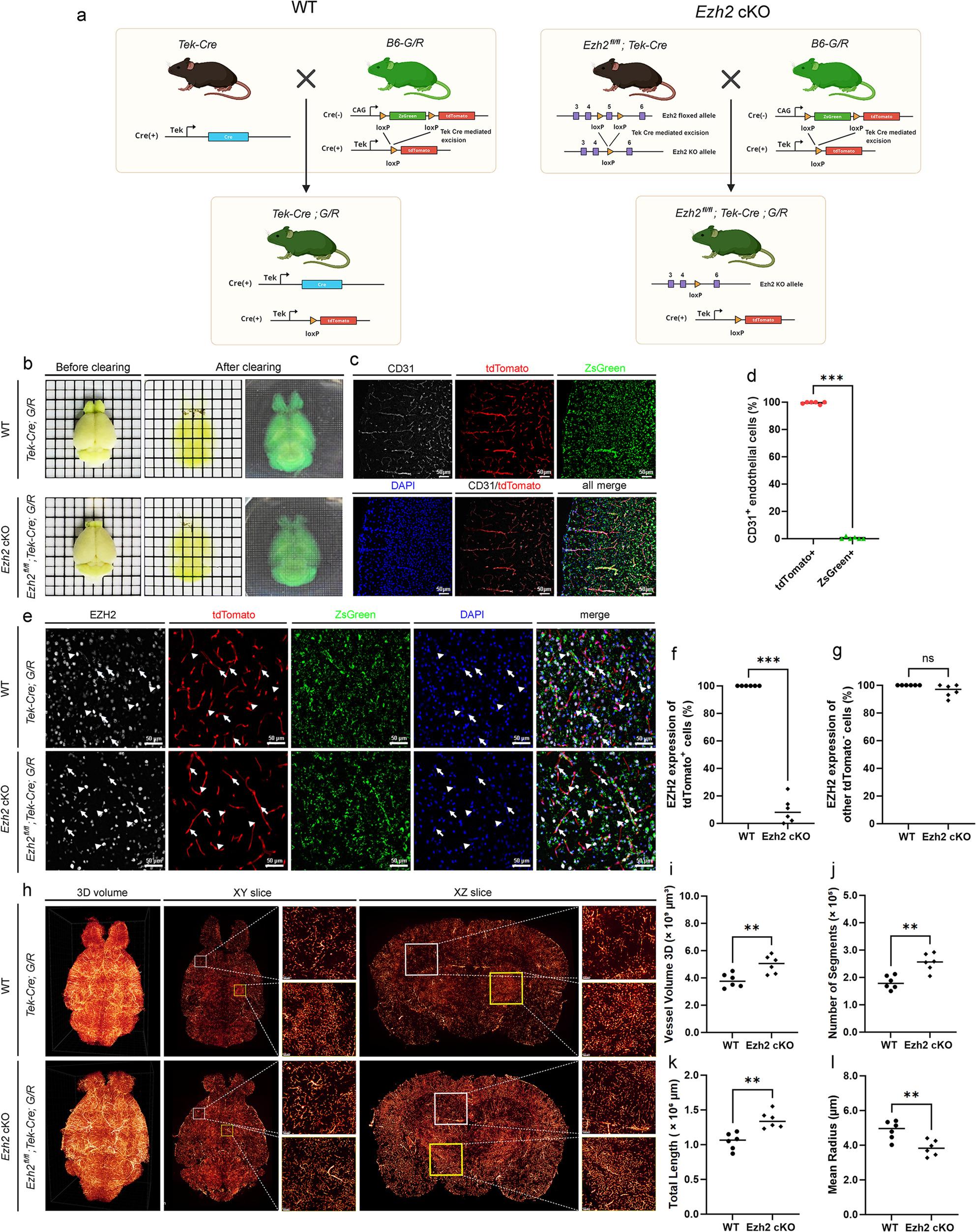
*Ezh2* Regulates Brain Vasculature Development and Integrity. **a** Genetic strategy for conditional knockout of *Ezh2* in endothelial cells. Top: Schematic representation of the breeding strategy to generate endothelial cell-specific *Ezh2* knockout mice (*Ezh2* cKO). *Ezh2* floxed mice were crossed with mice expressing Cre recombinase under the control of the Tek promoter (Tek-Cre), leading to deletion of *Ezh2* in endothelial cells. Bottom: Illustration of genetic recombination in WT (*Ezh2* ^flox/flox^; Tek-Cre negative) and *Ezh2* cKO (*Ezh2*^flox/flox^; Tek-Cre positive) mice. **b** Whole-brain clearing and visualization. Representative images of whole brains from WT and *Ezh2* cKO mice before and after tissue clearing. The transparent brains allow for deep tissue imaging of the vasculature. **c** Representative immunofluorescence images confirming endothelial cell-specific recombination in *Ezh2*^flox/flox^; Tek-Cre; G/R mice. Brain sections were stained for the endothelial cell marker CD31 (grey), tdTomato (red, indicating Cre-mediated recombination), and ZsGreen (green, indicating lack of Cre-mediated recombination in the *Ezh2* cKO model). DAPI (blue) counterstains nuclei. The ‘all merge’ panel shows the overlay of all channels. The tdTomato expression is robustly co-localized with CD31^+^ endothelial cells. And ZsGreen expression is minimal within CD31^+^ endothelial cells, indicating successful *Ezh2* deletion in these cells. Scale bar = 50 μm. **d** Quantification of tdTomato^+^ endothelial cells. Bar graph showing the percentage of CD31^+^ endothelial cells that are also tdTomato-positive in brains, confirming the efficiency and specificity of Cre-mediated recombination in endothelial cells. Data are presented as individual values with the mean. ***p < 0.001 (Mann-Whitney U test). **e** EZH2 expression in brain endothelial cells. Immunofluorescence images showing EZH2 expression (grey) and tdTomato (red) in endothelial cells (indicated by white arrows) or non-endothelial cells (indicated by white arrowheads) within brain sections from WT and *Ezh2* cKO mice. DAPI (blue) stains nuclei. Note the significant reduction of EZH2 signal in tdTomato-positive endothelial cells of *Ezh2* cKO mice and no change in non-endothelial cells. **f** Quantification of EZH2 expression in tdTomato^+^ cells. Bar graph quantifying the percentage of tdTomato-positive cells that also express EZH2, demonstrating the successful knockdown of *Ezh2* in endothelial cells of *Ezh2* cKO mice. Data are presented as mean ± SD. ****p < 0.001 (Mann-Whitney U test). **g** Specificity of *Ezh2* deletion. Bar graph showing the percentage of tdTomato-negative cells expressing EZH2, indicating that EZH2 expression is largely unaffected in non-endothelial cells in *Ezh2* cKO mice. Data are presented as individual values with the mean. ns: not significant (Student’s t-test). **h** 3D vascular reconstruction and analysis. Representative 3D volume renderings (left), XY slices (middle), and XZ slices (right) of brain vasculature from WT and *Ezh2* cKO mice, reconstructed from light-sheet microscopy data. White and yellow squares indicate regions magnified in the adjacent panels to show detailed vascular morphology. **i**-**l** Quantitative analysis of brain vasculature. Scatter plots as individual values with the mean showing the quantitative analysis of various vascular parameters: (**i**) Vessel Volume 3D (total volume occupied by vessels, µm³), (**j**) Number of Segments (count of individual vessel segments), (**k**) Total Length (total length of all vessels, µm), and (**l**) Mean Radius (average radius of vessels, µm) in WT and *Ezh2* cKO brains. Statistical significance was determined by Mann-Whitney U test. **p < 0.01; ***p < 0.0001

**Fig. 2:**
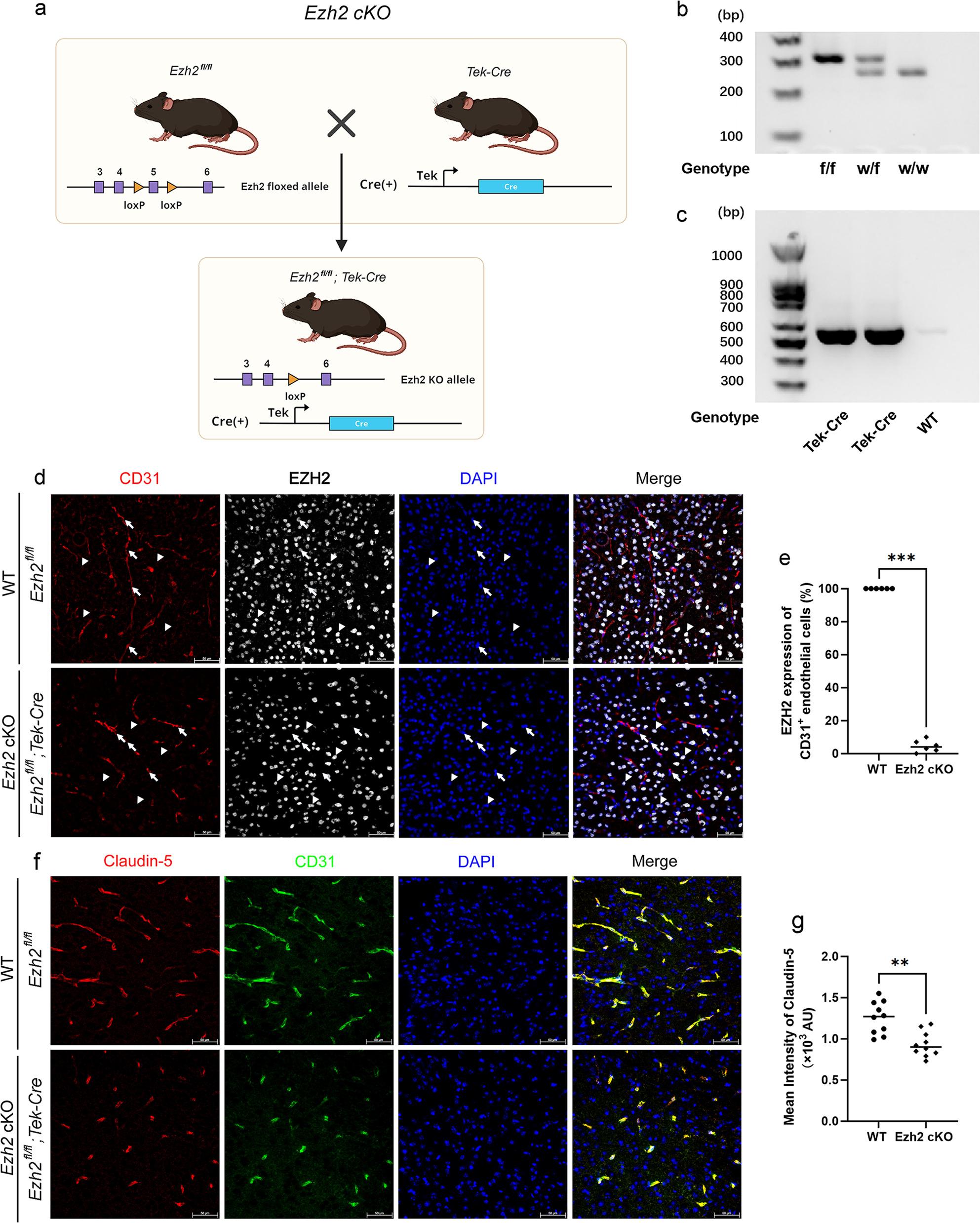
Endothelial-specific *Ezh2* Knockout in Mice and its Impact on Endothelial EZH2 Expression and Tight Junction Protein Claudin-5. **a** Generation of endothelial-specific *Ezh2* conditional knockout (cKO) mice. Schematic illustrating the breeding strategy to generate *Ezh2* cKO mice. *Ezh2* floxed mice (*Ezh2*^fl/fl^), carrying LoxP sites flanking the *Ezh2* gene, were crossed with mice expressing Cre recombinase under the control of the Tek (Tie2) promoter (Tek-Cre). This cross results in the deletion of *Ezh2* specifically in endothelial cells in the *Ezh2*^*fl/fl*^; Tek-Cre genotype. **b** Genotyping of *Ezh2* alleles by PCR. Representative gel electrophoresis image showing the PCR products for genotyping the *Ezh2* floxed allele (f/f) and the wild-type (w/w) allele. DNA from homozygous floxed (f/f), heterozygous (w/f), and wild-type (w/w) mice are shown. **c** Genotyping of Tek-Cre transgene by PCR. Representative gel electrophoresis image showing the PCR products for genotyping the Tek-Cre transgene. Bands indicate the presence of the Cre transgene in Tek-Cre positive mice, while its absence in WT mice is shown as a negative control. **d** Immunofluorescence staining for EZH2 in brain endothelial cells. Representative immunofluorescence images of brain sections from wild-type (WT) (*Ezh2*^fl/fl^) and *Ezh2* cKO (*Ezh2*^fl/fl^; Tek-Cre) mice. Sections were stained for CD31 (red), an endothelial cell marker; EZH2 (green), the target protein; and DAPI (blue), a nuclear stain. White arrows point to endothelial cells and white arrowheads point to non-endothelial cells. Note the robust EZH2 expression in CD31-positive endothelial cells in WT mice, and the significantly reduced or absent EZH2 signal in endothelial cells of *Ezh2* cKO mice. **e** Quantification of EZH2 expression in CD31^+^ endothelial cells. Bar graph representing the percentage of CD31^+^ endothelial cells that exhibit EZH2 expression in WT versus *Ezh2* cKO mice. Data are presented as individual values with the mean. ***p < 0.001 (Mann-Whitney U test), demonstrating efficient knockout of *Ezh2* in endothelial cells of cKO mice. **f** Immunofluorescence staining for Claudin-5 in brain vasculature. Representative confocal microscopy images of brain sections from WT and *Ezh2* cKO mice, stained for Claudin-5 (red), a key tight junction protein, and CD31 (green). DAPI (blue) stains nuclei. Note the change in Claudin-5 staining patterns in the vasculature of *Ezh2* cKO mice compared to WT. **g** Quantification of mean intensity of Claudin-5. Scatter plot as individual values showing the mean fluorescence intensity of Claudin-5 staining in the brain vasculature of WT and *Ezh2* cKO mice. Each dot represents an individual mouse. **p < 0.01 (Mann-Whitney U test), indicating a significant alteration in Claudin-5 expression or localization in the absence of endothelial *Ezh2*

**Fig. 3:**
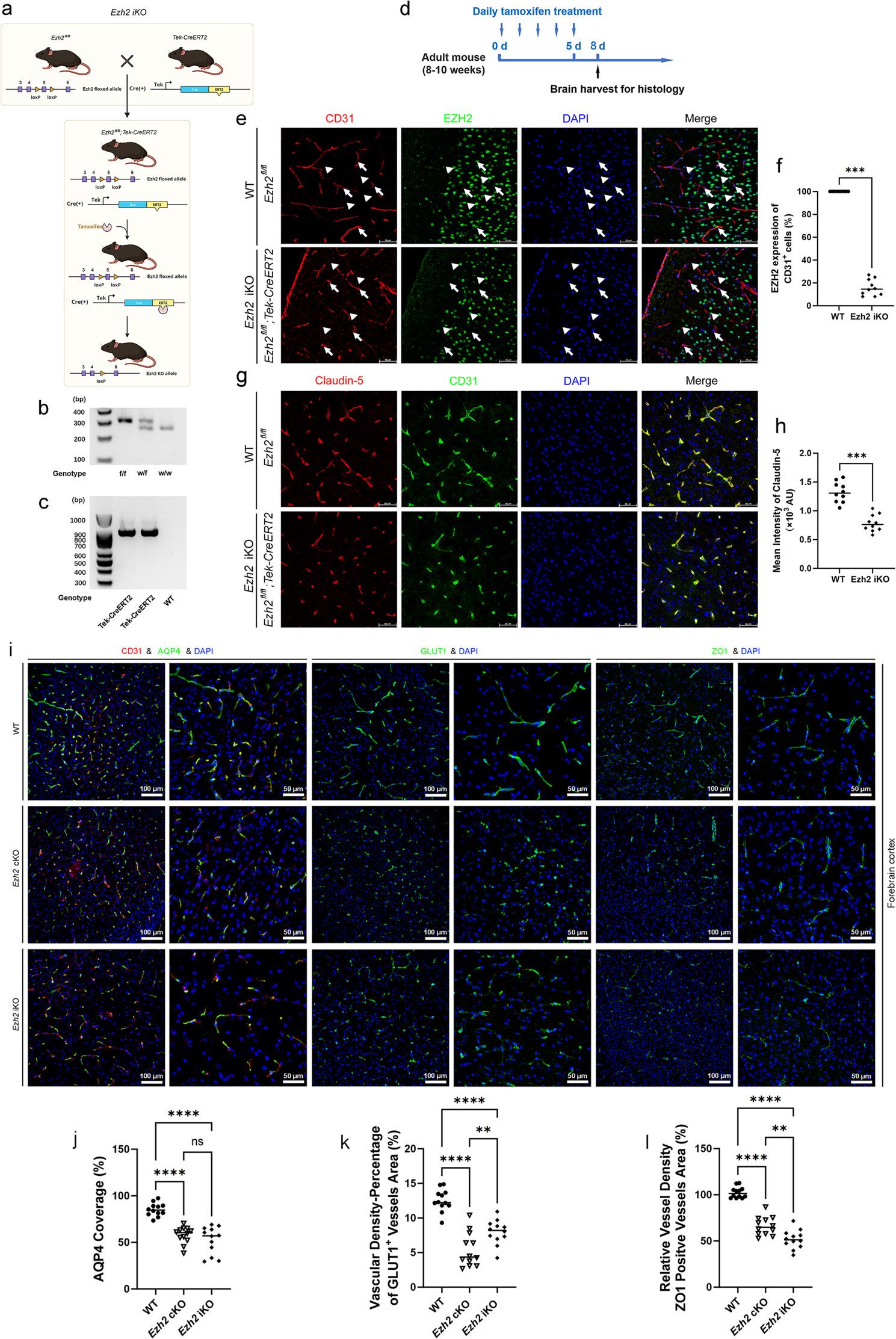
Endothelial *Ezh2* is essential for the maintenance of blood-brain barrier integrity and vascular density in the adult mouse brain. **a** Genetic strategy for inducible conditional knockout of *Ezh2* in endothelial cells. Top: Schematic representation of the breeding strategy to generate tamoxifen-inducible, endothelial cell-specific *Ezh2* knockout mice (*Ezh2* iKO). *Ezh2* floxed mice (*Ezh2*^fl/fl^) were crossed with mice expressing a tamoxifen-inducible Cre recombinase under the control of the Tek (Tie2) promoter (Tek-CreERT2). Bottom: Illustration of the genetic recombination process. Upon tamoxifen administration, CreERT2 translocates to the nucleus, leading to the excision of the *Ezh2* gene specifically in endothelial cells. **b** Genotyping of *Ezh2* alleles by PCR. Representative gel electrophoresis image showing the PCR products for genotyping the *Ezh2* floxed allele (f/f) and the wild-type (w/w) allele. DNA from homozygous floxed (f/f), heterozygous (w/f), and wild-type (w/w) mice are shown. **c** Genotyping of Tek-CreERT2 transgene by PCR. Representative gel electrophoresis image showing the PCR products for genotyping the Tek-CreERT2 transgene. Bands indicate the presence of the CreERT2 transgene in Tek-CreERT2 positive mice, while its absence in WT mice is shown as a negative control. **d** Tamoxifen treatment regimen. Timeline illustrating the daily tamoxifen treatment protocol for adult mice (8–10 weeks old). Tamoxifen was administered for 5 consecutive days, followed by a 2-day recovery period, and then brains were harvested for histological analysis. **e** Immunofluorescence staining for EZH2 in brain endothelial cells after tamoxifen induction. Representative confocal microscopy images of brain sections from wild-type (WT) (*Ezh2*^fl/fl^) and *Ezh2* iKO (*Ezh2*^fl/fl^; Tek-CreERT2) mice after tamoxifen treatment. Sections were stained for CD31 (red), an endothelial cell marker; EZH2 (green), the target protein; and DAPI (blue), a nuclear stain. White arrows point to endothelial cells and white arrowheads point to non-endothelial cells. Note the robust EZH2 expression in CD31-positive endothelial cells in WT mice, and the significantly reduced or absent EZH2 signal in endothelial cells of *Ezh2* iKO mice post-tamoxifen treatment. **f** Quantification of EZH2 expression in CD31^+^ endothelial cells. Bar graph representing the percentage of CD31^+^ endothelial cells that exhibit EZH2 expression in WT versus *Ezh2* iKO mice following tamoxifen induction. Data are presented as individual values with the mean. ***p < 0.001 (Mann-Whitney U test), demonstrating efficient inducible knockout of *Ezh2* in endothelial cells of iKO mice. **g** Immunofluorescence staining for Claudin-5 in brain vasculature. Representative confocal microscopy images of brain sections from WT and *Ezh2* iKO mice after tamoxifen treatment, stained for Claudin-5 (red), a key tight junction protein, and CD31 (green). DAPI (blue) stains nuclei. Note the change in Claudin-5 staining patterns in the vasculature of *Ezh2* iKO mice compared to WT after *Ezh2* deletion. **h** Quantification of mean intensity of Claudin-5. Scatter plot as individual values showing the mean fluorescence intensity of Claudin-5 staining in the brain vasculature of WT and *Ezh2* iKO mice. ***p < 0.001 (Mann-Whitney U test). **i** Representative confocal images of the forebrain cortex stained for vascular and perivascular markers: CD31/AQP4 (astrocyte endfeet), GLUT1 (glucose transporter 1), and ZO1 (tight junction protein 1). Comparisons are shown between WT, *Ezh2* cKO (constitutive), and *Ezh2* iKO (inducible) mice. Scale bars = 100 μm (low mag) and 50 μm (high mag). **j**-**l** Quantitative analysis of vascular parameters: (**j**) percentage of AQP4 coverage on vessels, (**k**) vascular density based on GLUT^+^ vessel area (%), and (**l**) relative vessel density based on ZO1^+^ area (%). Data reveal significant vascular rarefaction and loss of barrier-associated markers in both cKO and iKO models compared to WT. Data are presented as individual values with the mean. Statistical significance was determined by Mann-Whitney U test (**f**, **h**) or one-way ANOVA with Tukey’s post hoc test (**j**–**l**). **p < 0.01, ****p < 0.0001; ns, non-significant

To generate endothelial-specific *Ezh2* knockout mice, we utilized a Cre-LoxP system. Homozygous *Ezh2* flox mice (NM-CKO-190022) were crossed with Tek-Cre mice (Jackson Laboratory, Stock No: 008863) to generate conditional endothelial *Ezh2* knockout (*Ezh2* cKO) mice, designated as *Ezh2*^fl/fl^; Tek-Cre (Figs. 1a and 2a). For inducible endothelial *Ezh2* knockout (*Ezh2* iKO) mice, homozygous *Ezh2* flox mice were crossed with Tek-CreERT2 mice (Jackson Laboratory, Stock No: 030597) to generate *Ezh2*^fl/fl^; Tek-CreERT2 mice (Fig. 3a). For *Ezh2*^fl/fl^; Tek-Cre; G/R mice, the B6-G/R mice (T006163) were crossed with *Ezh2*^fl/fl^; Tek-Cre mice.

Wild-type (WT) littermates *Ezh2*^fl/fl^ without Cre were used as controls for all experiments. Both male and female mice, aged 8–12 weeks, were used in the studies and randomly assigned to experimental groups. Animals were housed in a temperature-controlled facility with a 12-hour light/dark cycle and provided with ad libitum access to food and water.

**Fig. 4:**
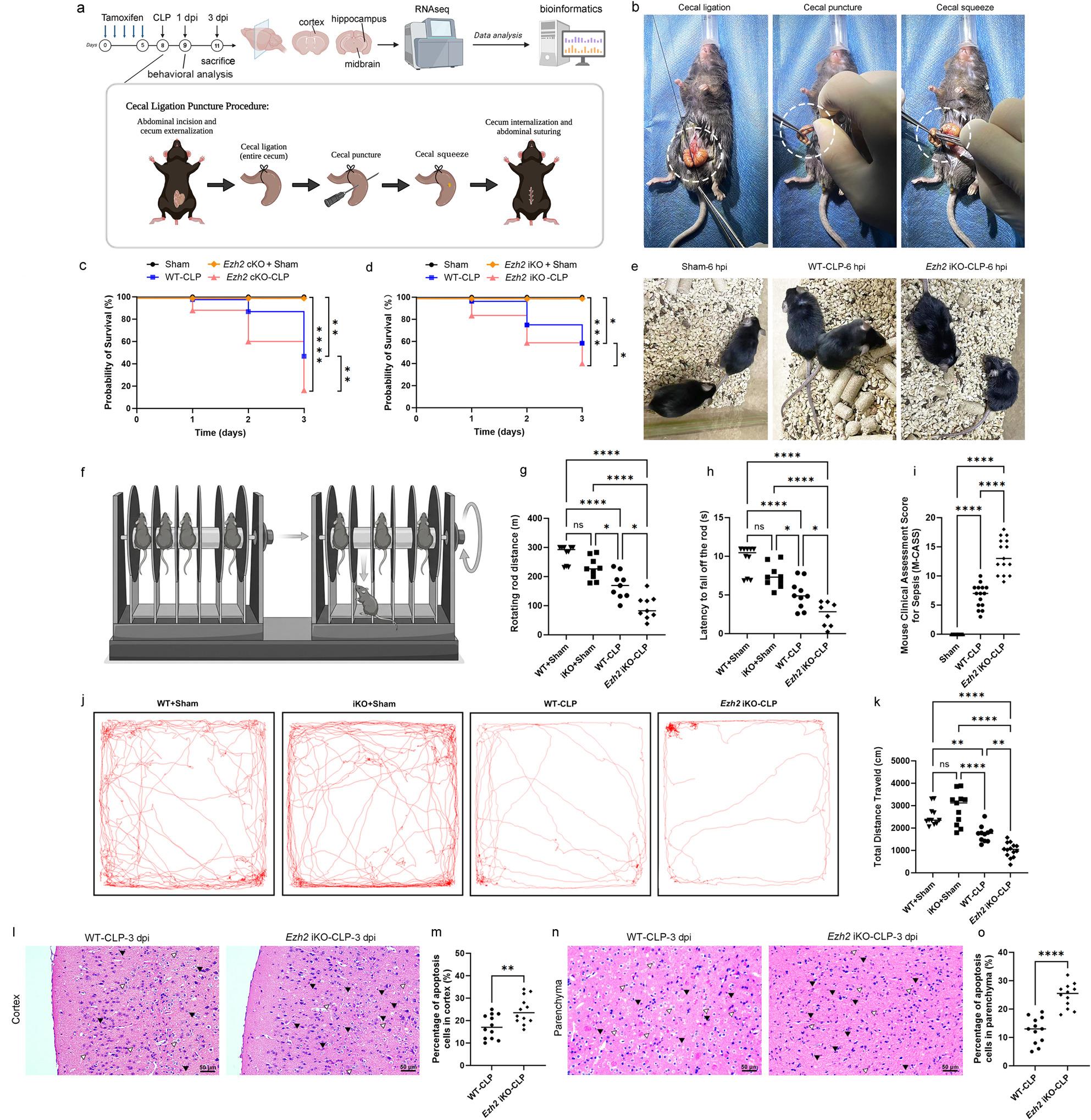
Endothelial *Ezh2* deficiency exacerbates sepsis-induced mortality, neurological impairment, and brain injury. **a **Schematic workflow of the experimental design. Adult mice received tamoxifen for inducible *Ezh2* deletion, followed by Cecal Ligation and Puncture (CLP) surgery. Behavioral assessments were performed prior to sacrifice at 3 days post-infection (dpi) for RNA sequencing (RNA-seq) of the cortex, hippocampus, and midbrain. **b** Representative surgical photographs illustrating the CLP procedure: ligation of the cecum, puncture with a needle, and extrusion of cecal contents (squeeze). **c**, **d** Kaplan-Meier survival curves comparing Sham, *Ezh2* cKO+Sham, *Ezh2* iKO+Sham, WT-CLP, and (**c**) *Ezh2 *cKO-CLP or (**d**) *Ezh2* iKO-CLP mice over 3 days. Loss of *Ezh2* significantly reduces survival probability following polymicrobial sepsis (n=12 per group). **e **Representative clinical photographs of mice 6 hours post-induction (hpi), showing increased lethargy and hunched posture in *Ezh2* iKO-CLP mice compared to WT-CLP and Sham controls. **f** Schematic of the rotarod test used to evaluate motor coordination and balance. **g**, **h** Quantification of rotarod performance, including (**g**) rotating rod distance (**m**) and **h** in *Ezh2* iKO-CLP mice. **i** Mouse Clinical Assessment Score for Sepsis (M-CASS) at 3 dpi, indicating increased disease severity in the iKO group. **j** Representative track maps from the open field test (OFT) and **k** Quantification of total distance traveled (cm), showing reduced locomotor activity and exploratory behavior in *Ezh2* iKO-CLP mice. **l**, **n** Representative images of H&E-stained brain sections highlighting the (**l**) cortex and **n** parenchyma at 3 dpi. Arrowheads indicate apoptotic/pyknotic nuclei (condensed, fragmented nuclei) characteristic of cell death. **m**, **o** Quantification of the percentage of apoptotic cells in the (**m**) cortex and **o** parenchyma, demonstrating increased neurodegeneration in the absence of endothelial *Ezh2*. Data are presented as individual values with the mean. Statistical significance was determined by Log-rank (Mantel-Cox) test for survival (**c**, **d**), one-way ANOVA with Tukey’s post hoc test (**g**, **h**,** k**, **i**), or unpaired Student’s t-test (**m**, **o**). *p < 0.05, **p < 0.01, ****p < 0.0001; ns, non-significant. Scale bars = 50 μm

For inducible *Ezh2* deletion in *Ezh2*^fl/fl^; Tek-CreERT2 mice, tamoxifen (Sigma-Aldrich, T5648) was dissolved in corn oil (Shanghai Yuanye Bio-Technology Co., Ltd, S24927) at 20 mg/mL and administered by oral gavage at a dose of 100 mg/kg once daily for five consecutive days (Figs. 3d and 4a). Experiments were performed two days after the last tamoxifen administration.

### Genotyping

Mouse genomic DNA was extracted from toes clips using the One Step Mouse Genotyping Kit (PD101-01, Vazyme, Nanjing, China) according to the manufacturer’s instructions. PCR was performed to determine the presence of *Ezh2*^fl/fl^, Tek-Cre, and Tek-CreERT2 alleles. Primer sequences for genotyping were as follows. *Ezh2* floxed allele: F-GTGTTTAGAATGCTGGGCAAGTG; R- TTGACAACCAGAACTCAATCCCT. Tek Cre: F-CGCATAACCAGTGAAACAGCATTGC; R- CCCTGTGCTCAGACAGAAATGAGA. Tek CreERT2: F- GACCAGGTTCGTTCACTCA; R- CAAGTTAGGAGCAAACAGTAGC. B6G/R KI: F- CCTCCTCTCCTGACTACTCCCAGTC; R- TCACAGAAACCATATGGCGCTCC. B6G/R WT: F- CAGCAAAACCTGGCTGTGGATC; R- ATGAGCCACCATGTGGGTGTC. PCR products were separated on a 1.5% agarose gel and visualized with a UV transilluminator (Figs. 2b-c and 3b-c).

### Whole-brain clearing, imaging and cerebral vasculature analysis

Whole-brain clearing was performed using the Tissue Clearing Kit (NH210701, Nuohai Life Science (Shanghai) Co., Ltd.). Briefly, each fixed sample was immersed in 50 mL of Clarifying Solution 1 at 37 °C for 5 days with gentle shaking, and the Clarifying Solution I was refreshed every day. Each sample was then washed by immersion in PBS for 6 h with gentle shaking at room temperature, and the PBS was renewed every 2 h. Using an equal volume of Solution II incubate each sample for an additional 4 days at 37 °C with gentle shaking, then the samples were washed in PBS as described above. Cleared brains were imaged using a light-sheet microscope (Nuohai LS 18 Tiling Light Sheet Microscope, laser line: 561 nm). A 1-tile tiling light sheet was used to illuminate the sample [32], and a 1×/0.25NA objective (Olympus MVPLAPO) was used to collect the fluorescence. The magnification of the microscope was set at 1.25×, and the spatial resolution was roughly 10 × 10 × 20 µm^3^ at the selected imaging conditions.

The collected images were processed with the LS 18 ImageCombine software (Nuohai Life Science (Shanghai) Co., Ltd) and rendered using Amira (Thermo Fisher Scientific, USA). For detailed cerebral vasculature analysis (Fig. 1h-l), whole-brain clearing was performed as described above. 3D volume rendering and vascular segmentation were performed using Imaris software (Bitplane) or similar image analysis software. Vessel density, total length, number of segments, and mean radius were calculated from the segmented 3D vascular networks. At least 3 independent brains were analyzed per group.

### Immunofluorescence staining and microscopy

Mice were transcardially perfused with ice-cold PBS followed by 4% Paraformaldehyde (PFA) solution. Brains were harvested, post-fixed in 4% PFA overnight at 4 °C, and then cryoprotected in 30% sucrose solution at 4 °C until fully submerged. Brains were embedded in Optimal Cutting Temperature (O.C.T.) compound (Sakura Finetek, 4583) and snap-frozen. Coronal brain Sect.  (25 μm thick) were prepared using a cryostat (Thermo Scientific CryoStar NX70) and mounted onto Superfrost Plus slides.

Sections were permeabilized with 0.3% Triton X-100 in PBS for 15 min, blocked with 5% normal donkey serum in PBS for 1 h at room temperature, and then incubated overnight at 4 °C with primary antibodies: CD31 (Platelet Endothelial Cell Adhesion Molecule-1, PECAM-1) (Abcam, ab28364) for endothelial cells. EZH2 (e.g., Cell Signaling Technology, 5246 S). Claudin-5 (Thermo Fisher Scientific, 34-1600). GFAP (Glial Fibrillary Acidic Protein) (Abcam, ab4674) for astrocytes. Olig2 (Oligodendrocyte Transcription Factor 2) (MilliporeSigma, MABN50) for oligodendrocytes. IBA1 (Ionized Calcium-Binding Adapter Molecule 1) (Abcam, ab178847) for microglia. Anti-CD8 alpha rabbit mAb (Servicebio GB15068-50), anti-CD4 rabbit mAb (Servicebio, GB15064-50), anti-CD3 mouse mAb (Servicebio GB12014-50) for T cell, anti-ly6g rabbit pAb (Servicebio GB11229-50) for neutrophils, anti-aquaporin 4 mouse mAb (Servicebio, GB12529), anti-goat rCD31/PECAM-1 (R&D, AF3628), anti-albumin (Proteintech, 16475-1-AP), anti-glucose transporter GLUT1 Rabbit pAb (Servicebio, GB113495), anti-ZO1 tight junction protein rabbit pAb (Servicebio, GB111402), anti-cleaved caspase-3 rabbit mAb (Cell signaling, #9664). After primary antibody incubation, sections were washed thrice with PBS and incubated for 1 h at room temperature with appropriate species-specific secondary antibodies conjugated with Alexa Fluor 488, 555, or 647 (Thermo Fisher Scientific). Nuclei were counterstained with DAPI (Sigma-Aldrich, D9542).

Images were acquired using a Leica THUNDER Imager DMI8 fluorescence microscope (Leica Microsystems, Wetzlar, Germany). For quantitative analysis, at least 12 images were captured per animal from comparable brain regions (cortex, hippocampus, midbrain). Image acquisition settings were kept consistent between experimental groups. Quantification of fluorescent intensity and positive cells was performed using ImageJ software (NIH). For the quantitative analysis of neuroinflammation, the populations of astrocytes (GFAP^+^), microglia (IBA1^+^), and oligodendrocyte lineage cells (Olig2^+^) were determined using absolute cell density. Representative images were acquired using a confocal microscope with a consistent field of view (FOV). The total number of marker-positive cells was counted in at least five randomly selected, non-overlapping regions within the cortex and hippocampus (Bregma − 1.70 mm to -2.18 mm) per mouse. The area of each FOV was measured in square micrometers (µm^2^) using ImageJ software and subsequently converted to square millimeters (mm^2^). The cell density was calculated as the total number of positive cells divided by the total area of the analyzed region (cells/mm^2^). To maintain anatomical consistency, all measurements were restricted to defined anatomical boundaries. Mean intensity of Claudin-5 was calculated using ImageJ.

For the quantitative analysis of the choroid plexus, marker-positive cells were counted in the choroid plexus of both lateral ventricles and fourth ventricle using standard coronal sections (Bregma − 1.70 mm to -2.18 mm). All measurements were restricted to defined anatomical boundaries of the ventricular space .

### Cecal Ligation and Puncture (CLP) Model

The CLP model was adapted from established protocols to induce sepsis in mice (Fig. 4a-d). Mice were anesthetized with isoflurane. A midline abdominal incision was made, and the cecum was carefully exposed. The cecum was ligated with a 4 − 0 silk suture at 1/3 of its distal end, avoiding bowel obstruction. The ligated cecum was then punctured twice with a 21-gauge needle, allowing a small amount of fecal material to extravasate. The cecum was returned to the abdominal cavity, and the incision was closed in layers. Sham-operated mice underwent the same procedure without ligation or puncture. All mice received a subcutaneous injection of pre-warmed sterile saline (1 mL/20 g body weight) immediately after surgery for fluid resuscitation and buprenorphine (0.05 mg/kg) for analgesia. Survival was monitored for up to 3 days post-CLP. Brains were harvested at 3 days post-CLP for histological and molecular analyses.

### Behavioral analysis and Mouse Clinical Assessment Score for Sepsis (M-CASS)

Locomotor activity and exploratory behavior were assessed using the Open Field Test (OFT) [33]. Mice were placed in the center of a square gray plastic arena (40 × 40 × 40 cm) and allowed to explore freely for 10 min. An automated video tracking system (Any-maze, Stoelting Co., USA) was used to record the total distance traveled (m), mean speed (cm/s), and the time spent in the central zone (defined as the inner 20 × 20 cm area). A decrease in total distance indicates impaired motor function or lethargy, while reduced time in the central zone reflects increased anxiety-like behavior.

Motor coordination and balance were evaluated using an accelerating rotarod apparatus [34]. Prior to the CLP surgery, mice underwent a training period for three consecutive days, consisting of three trials per day at a constant speed (5 rpm) for 120 s. On the testing day (24 h post-CLP), mice were placed on the rod which accelerated from 4 to 40 rpm over a 5-minute period. The latency to fall (the time from the start of the trial until the mouse fell from the rod or made two full passive rotations) was recorded. Each mouse was given three trials with a 15-minute inter-trial rest interval, and the average latency to fall was calculated for statistical analysis.

To quantify the systemic severity and frailty of mice following CLP, a modified Mouse Clinical Sepsis Score (M-CASS) was implemented [35] (Table S1). Mice were evaluated at 6, 12, and 24 h post-surgery by two independent researchers blinded to the genotypes. The scoring system (0–12 points) assessed four key clinical parameters: (1) Appearance (0: smooth coat; 1: slight piloerection; 2: severe piloerection; 3: hunched posture); (2) Level of consciousness (0: active; 1: mild depression; 2: lethargic; 3: unresponsive); (3) Activity (0: normal; 1: reduced movement; 2: movement only after stimulation; 3: no movement); and (4) Response to stimulus (0: normal; 1: mild delay; 2: severely delayed; 3: no response). The cumulative score served as a measure of frailty and disease progression.

### Assessment of BBB permeability via evans blue leakage

To assess functional BBB integrity, mice received a tail vein injection of 2% Evans Blue dye (Sigma-Aldrich; 4 mL/kg body weight) 24 h post-CLP. The dye was allowed to circulate for 2 h to ensure sufficient distribution. Following circulation, mice were deeply anesthetized and transcardially perfused with ice-cold PBS to remove intravascular dye. Brains were harvested and weighed.

### Immunofluorescence analysis of albumin extravasation

To evaluate the leakage of endogenous blood-borne proteins into the brain parenchyma, 30 μm thick cryosections were used. Sections were blocked with 5% normal goat serum and incubated with Alexa Fluor 488-conjugated goat anti-albumin antibody (1:200; Proteintech, 16475-1-AP) overnight at 4 °C. Albumin’s presence outside the CD31 positive vasculature indicates significant BBB breakdown. Fluorescence intensity was quantified using ImageJ software across five random fields per section in the cortex. For the quantitative analysis of BBB permeability, high-resolution immunofluorescence images of albumin staining were analyzed using ImageJ. Briefly, the images were converted to 8-bit grayscale, and a constant threshold was applied to each section to eliminate background noise and isolate the extravasated signal. The area fraction (%) was calculated as the percentage of pixels above the threshold within a defined Region of Interest (ROI) in the cortex and hippocampus. To ensure accuracy, intravascular albumin signal was excluded by co-staining with the endothelial marker CD31, ensuring that the quantified area represents only protein that has leaked into the extravascular space.

### Histological staining (H&E and Toluidine Blue)

For Hematoxylin and Eosin (H&E) staining (Fig. 4l-o), brains were fixed in 10% neutral buffered formalin, embedded in paraffin, and cut into 5 μm thick sections. Sections were deparaffinized and rehydrated, followed by staining with hematoxylin and eosin according to standard protocols. Images were captured using a brightfield microscope (Leica DM300, Leica Microsystems, Wetzlar, Germany). Apoptotic cells were identified by characteristic morphological changes, including nuclear condensation and fragmentation, and quantified from at least 5 random fields per section, with 3 sections analyzed per animal. The percentage of apoptotic cells was calculated as (number of apoptotic cells / total number of cells) ×100.

**Fig. 5:**
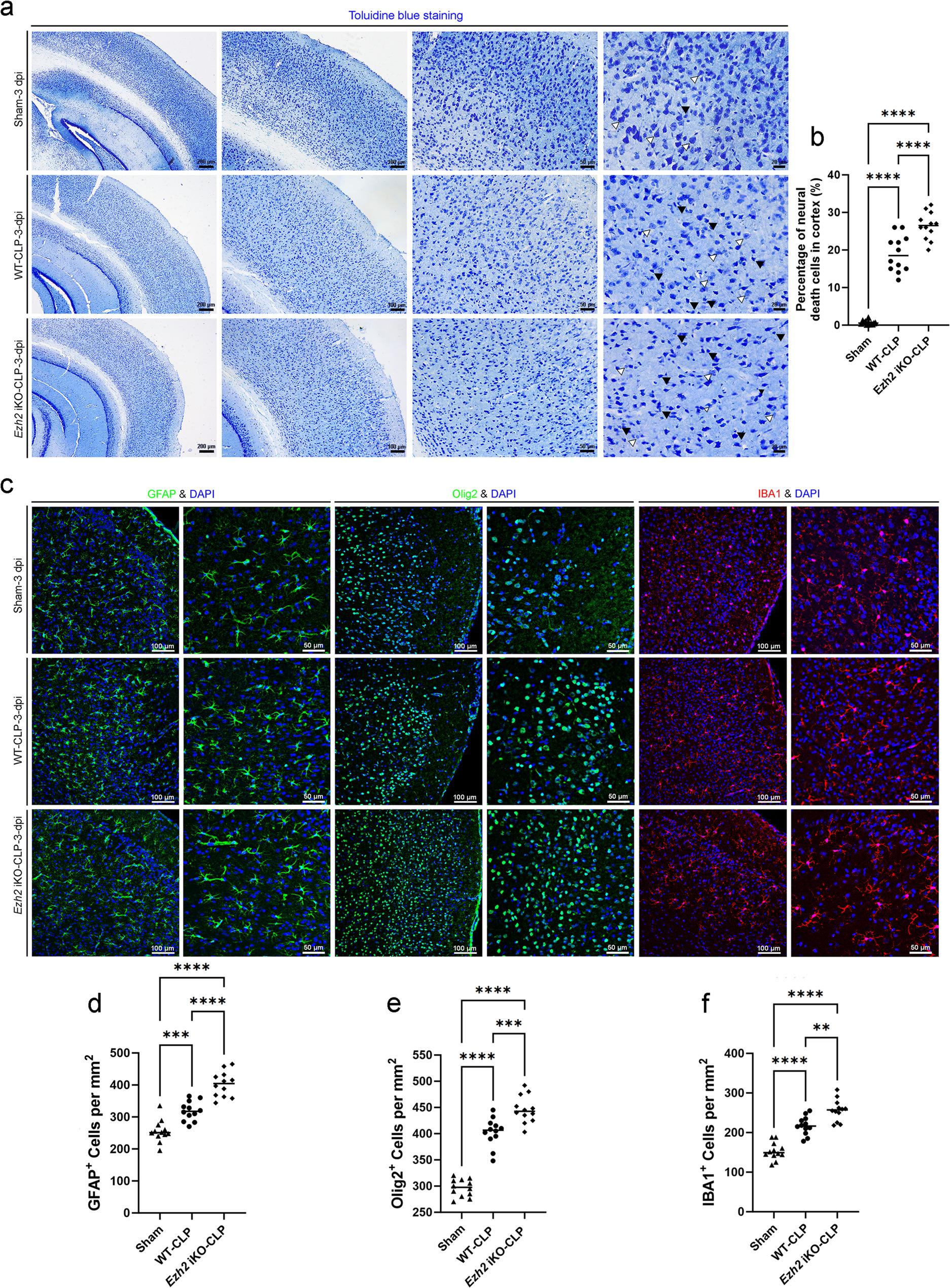
Endothelial *Ezh2* deficiency exacerbates neuronal damage and triggers robust glial activation in the septic brain. **a** Representative images of Toluidine Blue staining in the brain (cortex and hippocampus) at 3 dpi. Higher magnification panels reveal distinct morphological changes in Sham, WT-CLP, and *Ezh2* iKO-CLP groups. Black arrowheads highlight dark, shrunken, and degenerated neurons (pyknotic cells), while white arrowheads indicate healthy neurons. **b** Quantification of the percentage of neural death cells in the cortex, showing a significant increase in neuronal loss in *Ezh2* iKO-CLP mice compared to WT-CLP. **c** Representative confocal immunofluorescence images of the cortex stained for markers of glial activation: GFAP (astrocytes, green), Olig2 (oligodendrocytes, green), and IBA1 (microglia, red), with DAPI (nuclei, blue). **d**–**f** Quantitative analysis of glial cell densities: (**d**) GFAP^+^ cells per mm^2^, (**e**) Olig2^+^ cells per mm^2^, and (**f**) IBA1^+^ cells per mm^2^. The data demonstrate that sepsis-induced glial activation and proliferation are significantly exacerbated in the absence of endothelial *Ezh2*, suggesting a link between vascular dysfunction and neuroinflammation. Data are presented as individual values with the mean. Statistical significance was determined by one-way ANOVA with Tukey’s post hoc test. **p < 0.01, ***p < 0.001, ****p < 0.0001. Scale bars = 200 μm (low mag), 100 μm (medium mag), and 50 μm (high mag)

**Fig. 6:**
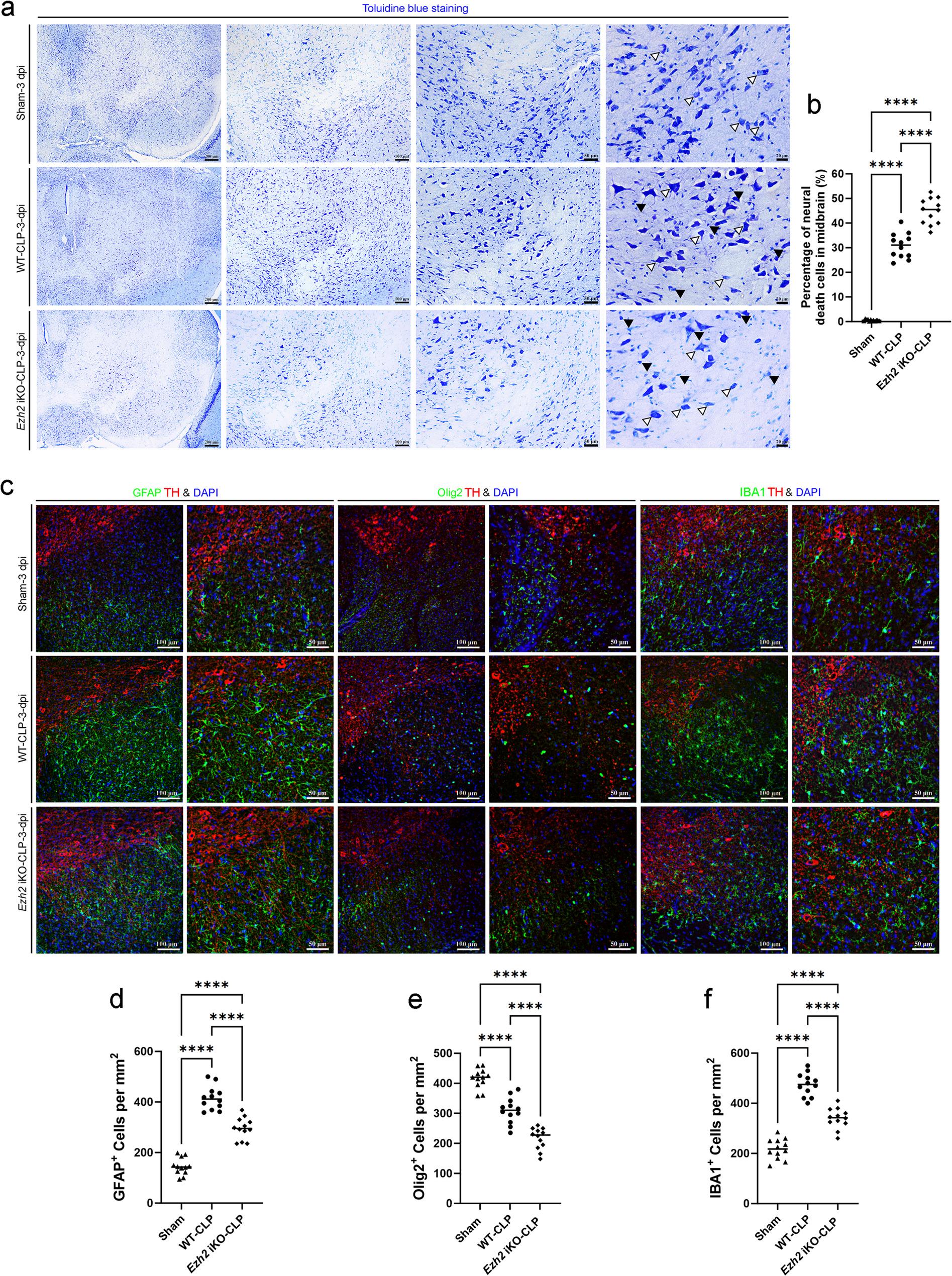
*Ezh2* deficiency exacerbates neuronal loss and modulates glial activation in the midbrain following sepsis-induced injury. **a** Representative images of Toluidine blue (Nissl) staining in the midbrain sections of Sham, WT-CLP, and *Ezh2* iKO-CLP groups at 3 days post-infection (dpi). Successive panels show increasing magnifications of the injured area. White arrowheads indicate healthy neurons with distinct Nissl bodies; black arrowheads indicate degenerating neurons characterized by pyknotic nuclei and cytoplasmic shrinkage. Scale bars = 200 μm, 100 μm, 50 μm, and 20 μm (left to right). **b** Quantitative analysis of the percentage of neural death cells in the midbrain across the three groups. Data show a significant increase in neuronal death in the *Ezh2* iKO-CLP group compared to the WT-CLP group. **c** Representative immunofluorescence images of the midbrain at 3 dpi. Brain sections were triple-stained for Tyrosine Hydroxylase (TH, red), DAPI (blue), and specific glial markers (green): GFAP (astrocytes, left), Olig2 (oligodendrocytes, middle), and IBA1 (microglia, right). Scale bars = 100 μm (low mag) and 50 μm (high mag). **d**-**f** Quantitative comparison of the number of (**d**) GFAP^+^ reactive astrocytes, (**e**) Olig2^+^ oligodendrocytes, and (**f**) IBA1^+^ activated microglia per mm^2^ in the midbrain. Note that while CLP induction triggers significant glial activation and oligodendrocyte loss, *Ezh2* deletion further modulates these populations compared to WT-CLP mice. Data are presented as individual values with the mean (*n* = 12 per group). Statistical significance was determined by one-way ANOVA followed by Tukey’s post hoc test, ****p < 0.0001

**Fig. 7:**
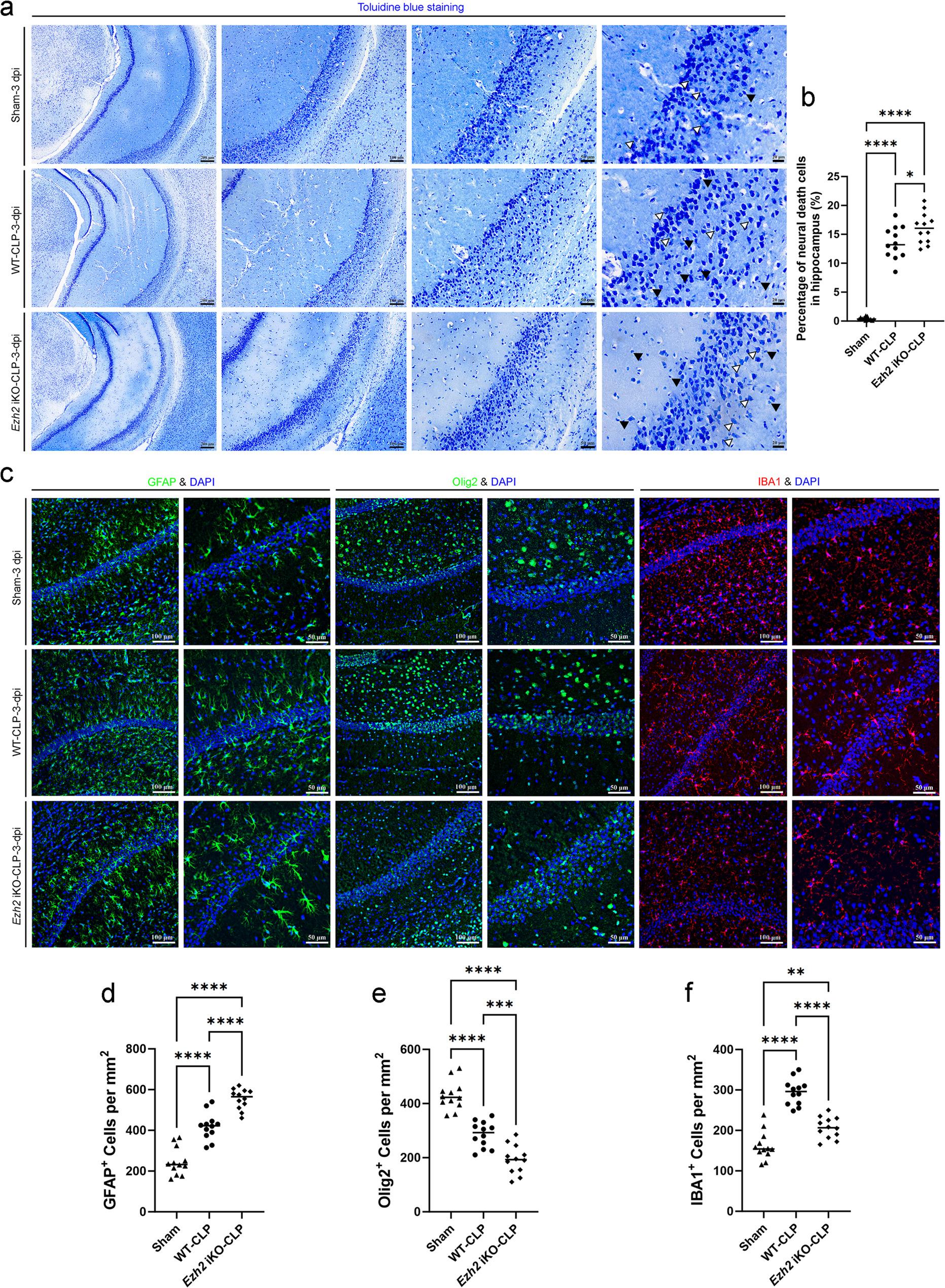
*Ezh2* deficiency exacerbates neuronal death and modulates glial responses in the hippocampus following sepsis-induced brain injury. **a** Representative images of Toluidine blue staining in the hippocampal region of Sham, WT-CLP, and *Ezh2* iKO-CLP mice at 3 days post-injury (dpi). Increasing magnifications (left to right) show the structural integrity of the hippocampal layers. Black arrowheads indicate damaged or pyknotic neurons characterized by shrunken cell bodies and condensed nuclei; white arrowheads indicate healthy neurons. Scale bars as indicated (200 μm, 100 μm, 50 μm, 25 μm). **b** Quantification of the percentage of neural death cells in the hippocampus. CLP significantly induces neuronal death, which is further exacerbated in the *Ezh2* iKO group. **c** Representative immunofluorescence images of the hippocampus at 3 dpi stained for glial markers (green or red) and counterstained with DAPI (blue) for nuclei. GFAP (green): Marker for astrocytes. Olig2 (green): Marker for oligodendrocytes. IBA1 (red): Marker for microglia/macrophages. Scale bars = 100 μm (low mag) and 50 μm (high mag). **d**-**f** Quantitative analysis of glial cell populations per mm^2^: (**d**) GFAP^+^ cells: CLP induces reactive astrogliosis, which is significantly increased in *Ezh2* iKO mice compared to WT-CLP mice. **e** Olig2^+^ cells: Sepsis-induced loss of oligodendrocytes is significantly more severe in the *Ezh2* iKO group compared to the WT-CLP group. **f** IBA1^+^ cells: While CLP increases microglial density in WT mice, this response is significantly attenuated in *Ezh2* iKO mice. Data are presented as individual values with the mean (*n* = 12 per group). Statistical significance was determined by one-way ANOVA followed by Tukey’s post hoc test. *p < 0.05, **p < 0.01, ***p < 0.001, ****p < 0.0001

For Toluidine blue staining (Figs. 5a, 6a and 7a), cryosections (25 μm) were fixed in 4% PFA, washed with PBS, and stained with 0.1% Toluidine blue solution in 1% sodium borate for 1–2 min. Sections were then rinsed with distilled water, dehydrated through an ethanol series, cleared in xylene, and mounted with a coverslip. Images were acquired using a brightfield microscope (Leica DM300). Neural death cells were identified by dark, condensed nuclei and overall neuronal shrinkage. Quantification of neural death cells was performed by counting positive cells in multiple fields of view and expressing them as a percentage of total cells.

### Immunofluorescence quantification and cell counting

For the quantitative assessment of neuronal apoptosis within the cortex, immunofluorescence images were analyzed using ImageJ/Fiji software (Version 1.54, NIH). Three to five representative fields of view (FOV) were randomly selected per section from a consistent region of interest (ROI) across all experimental groups. To ensure accuracy, images were subjected to background subtraction and synchronized thresholding for the NeuN (red) and Caspase3 (green) channels. The total number of NeuN-positive (NeuN^+^) neurons and Caspase3-positive (Caspase3^+^) cells were quantified using the “Analyze Particles” function. Apoptotic neurons were specifically identified by the colocalization of NeuN and Caspase3 signals. The final data were expressed as the percentage of apoptotic neurons, calculated using the formula: Apoptosis Rate (%) = (Number of NeuN^+^/Caspase3^+^ cells /Total number of NeuN^+^ cells) × 100. All counting and analysis were performed by an investigator blinded to the treatment groups to eliminate bias.

### RNA extraction and RNA Sequencing (RNA-seq)

Mice were transcardially perfused with ice-cold PBS. Brain regions (cortex, hippocampus, and midbrain) were dissected, flash-frozen in liquid nitrogen, and stored at -80 °C (Fig. 4a, Graphic Abstract). Total RNA was extracted using TRIzol reagent (Invitrogen, 15596026) following the manufacturer’s instructions. RNA quantity and quality were assessed using a NanoDrop spectrophotometer (Thermo Fisher Scientific) and an Agilent 2100 Bioanalyzer (Agilent Technologies). Transcriptome sequencing experiments and bioinformatics analysis of data were performed by the aid of NovelBio Bio-Pharm Technology Co., Ltd.

RNA sequencing libraries were prepared using the TruSeq RNA Library Prep Kit v2 (Illumina) according to the manufacturer’s protocol. Briefly, mRNA was purified using oligo(dT) beads, fragmented, and converted into cDNA. Adapters were ligated, and fragments were amplified by PCR. Libraries were validated on an Agilent 2100 Bioanalyzer and quantified by Quantitative Polymerase Chain Reaction. Sequencing was performed on an Illumina NovaSeq 6000 platform to generate 150 bp paired-end reads.

### Bioinformatic analysis of RNA-seq data

Raw sequencing reads were quality-checked using FastQC (v0.11.7) and trimmed using Trimmomatic to remove adapter sequences and low-quality reads. Clean reads were aligned to the mouse reference genome (mm10) using HISAT2 (v2.2.0) [36]. Gene expression levels were quantified using StringTie (v2.2.1), and gene counts were generated using featureCounts (v2.0.3) [37].

**Fig. 8:**
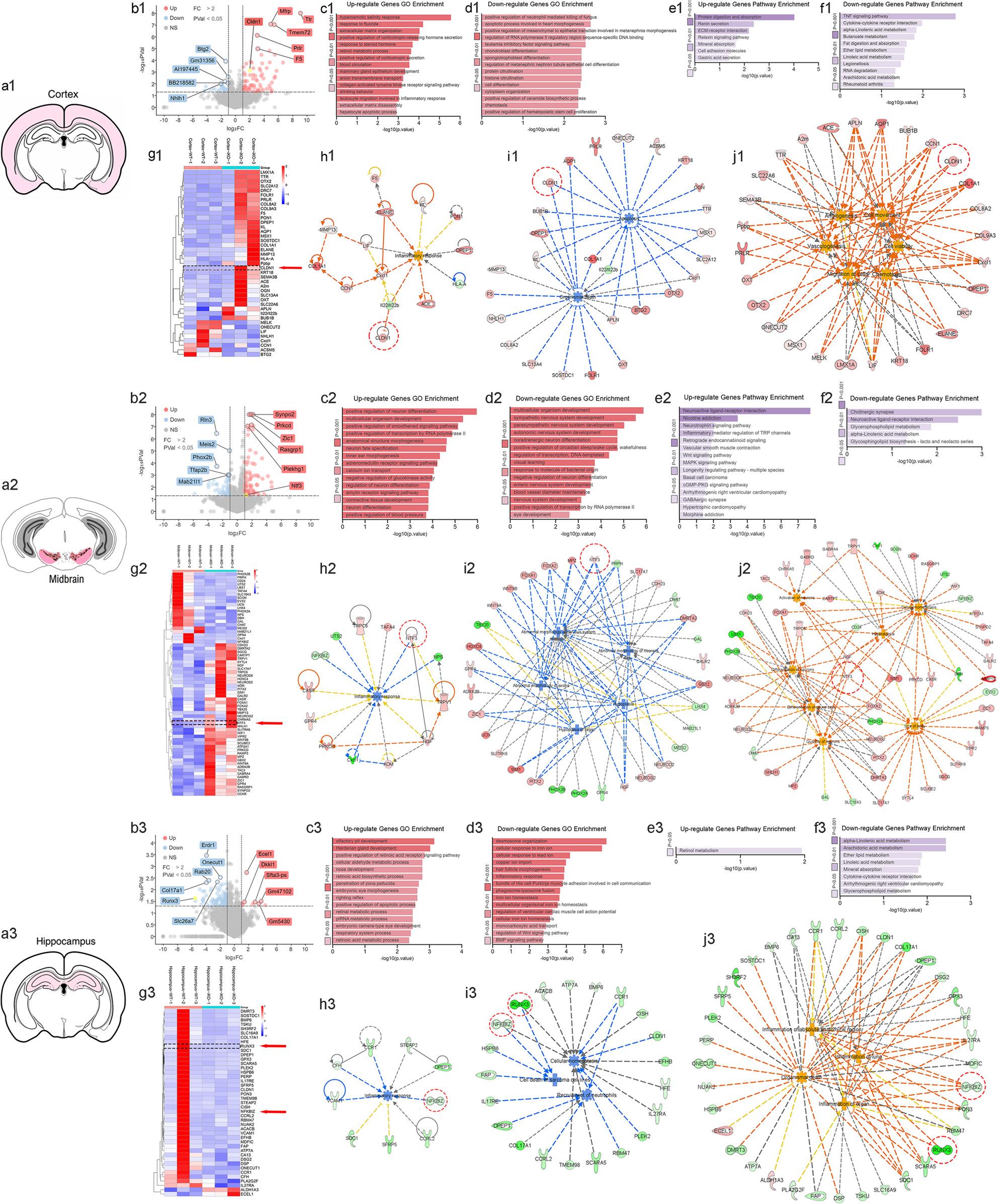
Comparative transcriptomic analysis across brain regions reveals regional-specific molecular signatures and regulatory networks mediated by EZH2 in the septic brain. (a1-a3) Schematic diagrams indicating the three brain regions harvested for RNA-seq analysis: (a1) Cortex, (a2) Midbrain, and (a3) Hippocampus. (b1-b3) Volcano plots representing differentially expressed genes (DEGs) in the (b1) cortex, (b2) midbrain, and (b3) hippocampus of *Ezh2* iKO-CLP mice compared to WT-CLP controls. Red and blue dots denote significantly up-regulated and down-regulated genes, respectively (Fold Change > 2, *p* < 0.05). Key genes such as *Cldn5*, *Tfn*, and *Slc2a1* are highlighted. (c1-c3, d1-d3) Gene Ontology (GO) enrichment analysis of (c1-c3) up-regulated and (d1-d3) down-regulated genes across the three regions. Enriched terms include “inflammatory response,” “response to cytokine,” “apoptotic process,” and “vascular development,” indicating widespread neurovascular and inflammatory dysregulation. (e1-e3, f1-f3) Pathway enrichment analysis for (e1-e3) up-regulated pathways and (f1-f3) down-regulated pathways, highlighting alterations in metabolic and signaling circuits. (g1-g3) Hierarchical clustering heatmaps showing the expression profiles of the top DEGs across individual replicates for each brain region. The consistency across replicates (Cortex iKO 1–3 vs. WT 1–3) demonstrates the robustness of the transcriptomic shift following *Ezh2* deletion. (h1-j3) Integrative network analysis (IPA) illustrating predicted regulatory interactions and hub gene networks in the (h1-j1) cortex, (h2-j2) midbrain, and (h3-j3) hippocampus. Networks highlight central nodes involved in (h) inflammatory signaling, (i) cell survival/death pathways, and (j) blood-brain barrier maintenance and cytoskeletal organization. Red-dashed circles identify key hub genes (e.g., *Cldn1*, *Mmp9*, *Tnf*) that drive the pathological phenotype in the absence of endothelial *Ezh2*

Differential gene expression analysis was performed using the DESeq2 package in R. Genes with an adjusted p-value (FDR); 0.05 and an absolute log2 fold change > 1 (corresponding to a 2-fold change) were considered significantly differentially expressed. Gene Ontology (GO) enrichment analysis and KEGG pathway analysis were performed using the CytoNavigator™ Data analytics platform, with significantly differentially expressed genes as input. Enriched terms and pathways with an adjusted *p*-value < 0.05 were considered statistically significant (Fig. 8, Graphic Abstract). Heatmaps of differentially expressed genes were generated using CytoNavigator™ Data analytics platform (Fig. 8, Graphic Abstract). Protein-protein interaction networks were constructed using IPA database and tools (IPA, QIAGEN, Redwood City) (Fig. 8, Graphic Abstract).

### Real-time quantitative RT-PCR

The quantification process involved a two-step reaction protocol, comprising reverse transcription (RT) and polymerase chain reaction (PCR). Reactions were carried out in a GeneAmp^®^ PCR System 9700 (Applied Biosystems, USA). Real-time PCR was subsequently executed using the LightCycler^®^ 480 Real-time PCR Instrument (Roche, Swiss). Each sample was subjected to triplicate analysis. At the conclusion of the PCR cycles, melting curve analysis was performed to validate the specific generation of the expected PCR product. The expression levels of mRNAs were then normalized to GAPDH and calculated using the2^−ΔΔCt^ method. Primer sequences were as follows. Cldn5-F GTTAAGGCACGGGTAGCACT; Cldn5-R TACTTCTGTGACACCGGCAC. Glut1-F GGATCCCAGCAGCAAGAAGGT; Glut1-R TAGCCGAACTGCAGTGATCC. Ocln-F CAGACCTGATGAATTCAAACCCA; Ocln-R AGAGTACGCTGGCTGAGAGA. Cdh5-F CGAACTGGATTCTCGGGGTAA; Cdh5-R TCGTAGGGCTGGGCAAATTC. Tjp1(ZO1)-F CCACCTCTGTCCAGCTCTTC; R CACCGGAGTGATGGTTTTCT.

### Statistical analysis

All quantitative data are presented as individual values with the mean, as indicated in figure legends. Statistical analyses were performed using GraphPad Prism software (version 9.0, GraphPad Software Inc., La Jolla, CA, USA). The Shapiro-Wilk test for normality and the Levene’s test (or F-test) for equality of variance for all datasets were performed. We screened all datasets using the Shapiro-Wilk test. For data that did not follow a normal distribution or exhibited unequal variances, we performed Mann-Whitney U test (for two-group comparisons). For comparisons among three or more groups, One-way or Two-way analysis of variance (ANOVA) followed by Tukey’s post-hoc test for multiple comparisons was performed. Survival curves were analyzed using the Kaplan-Meier method, and statistical significance was determined by the Log-rank (Mantel-Cox) test. A *p*-value < 0.05 was considered statistically significant. Specific statistical tests and *p*-values are indicated in the figure legends.

## Results

### Endothelial *Ezh2* deletion impairs blood-brain barrier integrity in mice

To investigate the role of endothelial *Ezh2* in maintaining BBB integrity, we generated endothelial-specific *Ezh2* cKO using a Cre-loxP system with Tek-Cre or Tek-CreERT2 drivers (Figs. 1a, 2a and 3a). The Tek-Cre mouse model facilitates conditional *Ezh2* deletion in endothelial cells, while the Tek-CreERT2 system allows for inducible deletion upon tamoxifen administration. Genotyping confirmed successful *Ezh2* deletion in these models (Figs. 2b-c and 3b-c, S1).

We first assessed the global clearing of brain tissue in *Ezh2* cKO mice. As shown in Fig. 1b, whole-brain clearing demonstrated a noticeable difference in tissue transparency between wild-type (WT) and *Ezh2* cKO mice after clearing, suggesting potential structural alterations. To specifically examine *EZH2* expression in endothelial cells, we performed immunofluorescence staining for CD31 (an endothelial cell marker), *EZH2*, and DAPI. In WT mice, *EZH2* was robustly expressed in CD31-positive endothelial cells, as evidenced by co-localization (Fig. 1c and e). However, in *Ezh2* cKO and iKO mice, there was a significant reduction in EZH2 expression within CD31^+^ endothelial cells (Figs. 1c and e, 2d and e and 3e and f), confirming the efficiency and specificity of our genetic deletion strategy.

Then, we quantitatively analyzed the cerebral vasculature using 3D volume rendering and subsequent segmentation. Compared to WT mice, *Ezh2* cKO brains exhibited a moderate increase in 3D vessel volume, number of segments, total vessel length, and reduction in mean radius (Fig. 1h-l). This indicates that endothelial *Ezh2* is not only crucial for the integrity of individual endothelial cells but also plays a vital role in maintaining the overall architecture and health of the cerebral vascular network. The alterations in vascular morphology, in conjunction with the decline in Claudin-5, collectively indicate a shift towards BBB characteristics in *Ezh2* cKO mice. Furthermore, the BBB is characterized by tight junctions between endothelial cells. To evaluate the impact of *Ezh2* deletion on tight junction integrity, we examined the expression of Claudin-5, a critical tight junction protein. Immunofluorescence staining revealed a marked decrease in Claudin-5 intensity in endothelial cells of *Ezh2* cKO mice compared to WT controls (Figs. 2f and g and 3g and h). This reduction in Claudin-5 expression strongly suggests impaired tight junction integrity in the absence of endothelial *Ezh2*, which could impair BBB function.

To further characterize the vascular defects, we analyzed additional markers of the neurovascular unit in the forebrain cortex, comparing WT mice with both constitutive *Ezh2* cKO and inducible *Ezh2* iKO models (Fig. 3i). We observed a marked decrease in the expression of the glucose transporter GLUT1, suggesting a compromise in the metabolic support capacity of the endothelium. Quantitative analysis confirmed a significant reduction in GLUT1 positive vascular density in both cKO and iKO cohorts (Fig. 3k). Similarly, the density of vessels positive for the tight junction protein ZO1 was significantly diminished following *Ezh2* deletion (Fig. 3l), further indicating a breakdown in the integrity of tight junctions and vessel organization.

The maintenance of the BBB is intrinsically linked to the interaction between endothelial cells and astrocytic endfeet. We assessed the coverage of Aquaporin-4 (AQP4), a water channel localized to the astrocytic endfeet surrounding the vasculature. In *Ezh2* iKO and cKO mice, we observed a significant reduction in AQP4 coverage compared to WT mice (Fig. 3j). These results suggest that endothelial EZH2 is not only required for the cell-intrinsic maintenance of tight junctions but is also essential for preserving the organizational integrity of the broader neurovascular unit in the adult brain.

To investigate the molecular consequences of *Ezh2* deletion, we quantified the mRNA expression levels of key endothelial and epithelial markers. Quantitative RT-PCR analysis revealed that the loss of *Ezh2* in both cKO and iKO models resulted in a significant downregulation of Glut1 and the tight junction protein ZO1 compared to WT controls (Figure S4a, S4b). Interestingly, while CDH5 expression was also significantly reduced in mutant mice (Figure S4c), the expression of Claudin-5 remained largely unaffected across all genotypes (Figure S4d), suggesting that EZH2 selectively regulates specific subsets of barrier-related genes rather than inducing a global transcriptional collapse. ZO1 was markedly diminished in the choroid plexus barrier of *Ezh2* cKO and iKO mice, with the iKO group displaying the most profound loss of signal (Figure S4e, S4f).

The vascular area was significantly reduced in both *Ezh2* mutant models, indicating a loss of microvascular density within the choroid plexus (Figure S4g). Double-labeling for GLUT1 and CD31 showed that *Ezh2* deficiency significantly lowered the GLUT1/CD31 ratio (Figure S4h), suggesting that the reduction in GLUT1 expression exceeds the overall loss of vascular surface area. EZH2 is required for maintenance of tight junctions, vascularization and GLUT1 expression in the choroid plexus, which may impact its blood-cerebrospinal fluid barrier function.

### *Ezh2* deficiency exacerbates systemic inflammation, reduces survival, and worsens neurological outcomes

Given the observed BBB impairment in *Ezh2* cKO mice, we hypothesized that these mice would be more susceptible to brain injury in conditions of systemic inflammation, such as sepsis. To test this, we employed a cecal ligation and puncture (CLP) model to induce sepsis in WT and *Ezh2* iKO mice (inducible knockout, using Tek-CreERT2 for tamoxifen-induced *Ezh2* deletion in endothelial cells) (Fig. 4a and b). We first assessed the survival rates over a 3-day period post-surgery. While the WT+Sham, *Ezh2* cKO+Sham and *Ezh2* iKO+Sham groups maintained 100% survival, the *Ezh2* cKO-CLP and *Ezh2* iKO-CLP cohorts exhibited significantly higher mortality compared to the WT-CLP group (Fig. 4c and d).

We performed intravenous Evans Blue (EB) injections (Figure S2a). Interestingly, while we did not observe gross, macroscopically visible blue staining of the brain parenchyma in most mice (Figure S2b, S2d). This finding is highly insightful, as it suggests that the BBB disruption in our model is not a gross, non-specific collapse (which would show macroscopic leakage), but rather a regulated increase in microvascular permeability. This indicates that *Ezh2* may play a novel role in vascular homeostasis that is distinct from total structural failure.

To evaluate the role of EZH2 in maintaining baseline brain fluid balance and gross structural integrity, we first examined brain morphology and wet weight in WT, *Ezh2* cKO, and *Ezh2* iKO mice under baseline conditions (Figure S2a). Representative gross anatomical images of harvested brains revealed no discernible morphological abnormalities across the three genotypes (Figure S2b). Quantitative analysis confirmed that the wet weight of the whole brain remained consistent among WT, *Ezh2* cKO, and *Ezh2* iKO mice, with no statistically significant differences observed (Figure S2c). These data indicate that EZH2 is not essential for the maintenance of gross brain structure or fluid balance in the absence of pathological challenge.

We next investigated the impact of *Ezh2* deficiency on the cerebral response to systemic inflammation via CLP model of sepsis. At 1 day post-surgery, all groups exhibited a significant increase in wet brain weight compared to baseline, indicative of sepsis-induced cerebral edema (Figure S2d, S2e). However, the severity of edema was markedly higher in *Ezh2*-deficient mice. Both *Ezh2* cKO and *Ezh2* iKO mice displayed significantly higher wet weights than WT-CLP mice (Figure S2e). These results suggest that EZH2 provides a protective effect against the accumulation of cerebral fluid during the acute phase of sepsis. We acknowledge that wet weight alone cannot distinguish between cerebral edema and other tissue components. The absence of dry weight measurements to calculate the wet-to-dry weight ratio is a limitation of this study. Future studies should utilize the drying method to precisely quantify the percentage of brain water content.

To determine if the observed increase in brain weight was due to compromised vascular permeability, we performed immunofluorescence staining for albumin extravasation and the endothelial marker CD31 at 3 dpi (Figure S2f). In Sham-operated mice, albumin was confined within the CD31-positive vasculature, representing a functional BBB. Following CLP, WT mice exhibited a moderate increase in albumin leakage into the brain parenchyma (Figure S2f). Notably, *Ezh2* iKO mice demonstrated a profound loss of barrier integrity, characterized by widespread albumin extravasation far beyond the vascular lumens (Figure S2f). Quantitative analysis of the albumin area fraction confirmed these observations: while WT-CLP mice showed an increase in leakage compared to Sham group, the leakage in *Ezh2* iKO-CLP mice was significantly more severe, reaching nearly triple the area fraction of WT-CLP controls (Figure S2g). Collectively, these findings demonstrate that EZH2 is a vital regulator of BBB stability, and its absence facilitates pathological permeability and subsequent edema during systemic inflammation.

Under CLP conditions, the reduction in GLUT1 expression and vascular integrity was further exacerbated in *Ezh2* cKO and iKO mice compared to WT-CLP controls (Figure S4i). Quantitative analysis confirmed a precipitous drop in the GLUT1/CD31 ratio in the mutant groups following inflammatory insult (Figure S4j). Notably, the *Ezh2* iKO-CLP group exhibited the most severe phenotype, suggesting that acute loss of *Ezh2* renders the choroid plexus barrier particularly vulnerable to inflammatory degradation.

### *Ezh2* loss aggravates sepsis-induced neurobehavioral impairments

Observation of sickness behavior at 6 h post-surgery revealed that *Ezh2* iKO mice displayed more pronounced lethargy and diminished exploratory activity compared to WT-CLP mice (Fig. 4e). These clinical observations were quantified using the Mouse Clinical Assessment Score for Sepsis (M-CASS), where *Ezh2* iKO-CLP mice scored significantly higher than their WT counterparts, indicating a more severe systemic disease state (Fig. 4i).

Given the systemic impact of *Ezh2* deficiency, we further evaluated its effects on neurological function and motor coordination, which are hallmarks of SAE. Using the rotarod test (Fig. 4i), we observed that while CLP itself reduced motor performance in WT mice, the deletion of *Ezh2* led to a more profound decline. Specifically, *Ezh2* iKO-CLP mice showed a significantly shorter rotating rod distance and reduced latency to fall compared to the WT-CLP group (Fig. 4g and h).

To assess locomotor activity and exploratory behavior, we conducted open field tests at 3 days post-surgery. Representative trajectory maps illustrated a marked reduction in movement within the *Ezh2* iKO-CLP group (Fig. 4j). Quantitative analysis confirmed that the total distance traveled was significantly lower in *Ezh2* iKO-CLP mice compared to WT-CLP mice (Fig. 4k), suggesting that EZH2 is essential for preserving neurological integrity during septic insult.

### Loss of *Ezh2* promotes brain damage and apoptosis

To determine the histopathological basis for the observed neurological deficits, we performed H&E staining and apoptotic cell quantification in the cortex and brain parenchyma. At 3 dpi, *Ezh2* iKO-CLP mice exhibited more severe morphological alterations and a higher density of shrunken, pyknotic nuclei compared to WT-CLP mice (Fig. 4l and n). Quantitative analysis revealed that *Ezh2* deficiency significantly increased the percentage of apoptosis in both the cortex (Fig. 4m) and the brain parenchyma (Fig. 4o). These findings indicate that EZH2 exerts a neuroprotective effect during sepsis by suppressing programmed cell death in critical brain regions.

Initial histological assessment using Toluidine blue staining revealed significant morphological changes in the forebrain cortex, midbrain and hippocampus of WT-CLP mice compared to the Sham group (Figs. 5a, 6a and 7a). In the WT-CLP group, we observed an increase in neurons exhibiting pyknotic nuclei and shrunken cell bodies, characteristic of neuronal degeneration. This damage was markedly more pronounced in the *Ezh2* iKO-CLP group. Quantitative analysis confirmed that while CLP-induced sepsis significantly increased the percentage of neural death cells in the forebrain cortex, midbrain and hippocampus, the loss of *Ezh2* further exacerbated this effect, leading to a significantly higher cell death rate compared to the WT-CLP group (Figs. 5b, 6b and 7b).

To further investigate the impact of EZH2 on neurodegeneration during SAE, we performed triple-immunofluorescence staining for NeuN, Caspase3, and DAPI across three critical brain regions: the cortex, midbrain, and hippocampus at 3 days post-surgery. Qualitative histological assessment (Figure S3a) revealed minimal Caspase3 immunoreactivity in the Sham-operated group across all observed regions, indicating negligible baseline apoptosis. Following the induction of sepsis via CLP, we observed a distinct increase in Caspase3-positive signals within NeuN-labeled neurons in WT mice, signifying sepsis-induced neuronal programmed cell death. Notably, this apoptotic phenotype was markedly intensified in the *Ezh2* iKO-CLP group. The density of Caspase3⁺/NeuN⁺ cells appeared substantially higher in the *Ezh2*-deficient septic mice compared to their WT septic counterparts, particularly in the cortical layers and the hippocampal formation.

Quantitative analysis corroborated these histological observations. In the cortex (Figure S3b), the percentage of Caspase3⁺/NeuN⁺ cells rose from baseline levels in the Sham group to approximately 20% in the WT-CLP group. However, conditional deletion of *Ezh2* resulted in a further, significant escalation of neuronal apoptosis, reaching approximately 35% (vs. WT-CLP). Similar trends were observed in the midbrain (Figure S3c) and hippocampus (Figure S3d). In the midbrain, *Ezh2* deficiency led to a nearly two-fold increase in the apoptotic index compared to WT septic mice. In the hippocampus-a region highly sensitive to inflammatory insult-the percentage of apoptotic neurons in the *Ezh2* iKO-CLP group was significantly elevated compared to the WT-CLP group, suggesting that EZH2 plays a pivotal neuroprotective role in maintaining neuronal survival during the acute phase of systemic sepsis. Taken together, these data demonstrate that the loss of *Ezh2* function significantly sensitizes neurons to sepsis-induced injury, leading to exacerbated apoptotic cell death across multiple brain regions, although it is also possible that *Ezh2* deletion may have similar effects in the absence of sepsis.

### Endothelial *Ezh2* deficiency promotes reactive gliosis and neuroinflammation in sepsis-associated encephalopathy

BBB disruption and neuronal death are often accompanied by reactive gliosis, which involves the activation of astrocytes and microglia. We investigated the activation of these glial cells in the brains of WT-CLP and *Ezh2* iKO-CLP mice. Immunofluorescence staining for GFAP (astrocyte marker), Olig2 (oligodendrocyte marker), and IBA1 (microglia marker) was performed.

In the forebrain cortex, midbrain and hippocampus, *Ezh2* iKO-CLP and WT-CLP mice exhibited a significantly higher percentage of GFAP-positive astrocytes compared to mice in sham group (Figs. 5c and d, 6c and d and 7c and d). This indicates widespread astrocytic activation, a common response to brain injury and inflammation. However, there were regional differences in astrocytic response among multiple brain regions. As demonstrated in all the forebrain cortex, midbrain and hippocampus (Figs. 5c and e, 6c and e and 7c and e), Olig2-positive cells exhibited a substantial augmentation in mice subjected to either *Ezh2* iKO-CLP or WT-CLP, in comparison to the sham group. The regional differences in oligodendrocyte response could reflect distinct vulnerabilities or regenerative capacities of different brain regions to *Ezh2* deficiency and sepsis, although it is also possible that *Ezh2* deletion may have similar effects in the absence of sepsis. We also observed a significant increase in IBA1-positive microglia in the forebrain cortex, midbrain and hippocampus of *Ezh2* iKO-CLP mice compared to WT-CLP mice (Figs. 5c and f, 6c and f and 7c and f). This indicates enhanced microglial activation, suggesting a heightened neuroinflammatory response in the cortex of septic *Ezh2*-deficient mice.

The increased activation of both astrocytes and microglia in *Ezh2* iKO-CLP mice further underscores the severity of neuroinflammation and brain injury in the absence of endothelial *Ezh2*. The implications of the above-described regional differences in astrocyte, oligodendrocyte and microglia response warrant further investigation.

To investigate the impact of endothelial *Ezh2* on the BBB and neuroinflammatory landscape during sepsis, we performed immunofluorescence analysis on the forebrain cortex and basal ganglia at 3 days post-surgery. In the forebrain cortex, CLP-induced sepsis resulted in a significant reduction of the CD31 positive microvascular area, an effect that was further exacerbated in *Ezh2* iKO mice (Figure S5a, b), suggesting that *Ezh2* is required for maintaining cerebrovascular structural integrity. This vascular rarefaction was accompanied by a robust increase in the infiltration of Ly6G positive neutrophils, with the *Ezh2* iKO-CLP group exhibiting significantly higher neutrophil density compared to WT-CLP controls (Figure S5c).

Beyond innate immune recruitment, we assessed the infiltration of adaptive immune cells, which are hallmark indicators of severe BBB disruption. Quantitative analysis of the forebrain cortex revealed that endothelial loss of *Ezh2* dramatically increased the recruitment of CD3 positive T cells, comprising both CD4 positive helper and CD8 positive cytotoxic T-cell subsets (Figure S5d-g). To determine if this neuroinflammatory phenotype was region-specific, we examined the basal ganglia. Consistent with our findings in the cortex, *Ezh2* iKO mice displayed a profound elevation in CD3 positive, CD4 positive, and CD8 positive T-cell infiltration following CLP challenge compared to their WT counterparts (Figure S5h-k). Collectively, these data demonstrate that endothelial *Ezh2* serves a protective role in the CNS during systemic inflammation by limiting pathological leukocyte infiltration and preserving the cerebral microvasculature.

We also characterized the infiltration of T lymphocyte subsets into the choroid plexus, a primary entry point for immune cells into the central nervous system. Using immunofluorescence staining at 3 days post-surgery, we observed that while CD3 positive T cells were sparse in the choroid plexus of both WT and *Ezh2* iKO Sham-operated mice, CLP induced a robust recruitment of T cells to this interface (Figure S6a). Quantitative analysis revealed that the density of infiltrating CD3 positive total T cells was significantly higher in *Ezh2* iKO-CLP mice compared to WT-CLP controls (Figure S6b). This increase was reflected across both major T cell lineages. Specifically, *Ezh2* deficiency led to a nearly two-fold increase in the accumulation of CD8 positive cytotoxic T cells (Figure S6c) and CD4 positive helper T cells (Figure S6d) within the ChP stroma following septic challenge. Notably, no significant differences were observed between WT and *Ezh2* iKO mice under sham conditions, suggesting that *Ezh2* is not required for homeostatic immune surveillance at the blood-CSF barrier but specifically functions to restrain excessive T cell recruitment during systemic inflammation.

### Transcriptomic analysis reveals dysregulated pathways in endothelial *Ezh2* deficiency during sepsis

To gain a deeper understanding of the molecular mechanisms underlying the exacerbated brain injury in *Ezh2* iKO-CLP mice, we performed RNA sequencing on brain tissue from WT-CLP and *Ezh2* iKO-CLP mice (Figs. 4a and 8, 8a1-a3, Graphic Abstract). Differential gene expression analysis revealed significant transcriptomic changes.

Volcano plots highlighted numerous up-regulated and down-regulated genes in *Ezh2* iKO-CLP brains compared to WT-CLP (Fig. 8b1-b3, Graphic Abstract). Gene Ontology (GO) enrichment analysis of up-regulated genes in the cortex revealed enrichment in pathways related to immune response, inflammatory response, and cell death (Fig. 8c1-c3, Graphic Abstract). Conversely, down-regulated genes were enriched in pathways associated with synaptic function, neuronal development, and metabolic processes (Fig. 8d1-d3, Graphic Abstract). Similar patterns were observed in the midbrain and hippocampus.

Pathway enrichment analysis (KEGG pathways) further supported these findings, identifying significant dysregulation in pathways crucial for maintaining brain homeostasis. Specifically, inflammatory signaling pathways, such as TNF signaling and NF-κB signaling, were significantly up-regulated (Fig. 8e1-e3, 8f1-f3, Graphic Abstract). This indicates a highly pro-inflammatory environment in the brains of *Ezh2* iKO-CLP mice during sepsis.

Heatmaps of differentially expressed genes provided a visual representation of the altered gene expression profiles (Fig. 8g1-g3, Graphic Abstract). Network analysis further elucidated potential interactions between these dysregulated genes and their involvement in critical biological processes (Fig. 8h1-h3, 8i1-i3, 8j1-j3, Graphic Abstract). These transcriptomic data provide compelling evidence that endothelial *Ezh2* deficiency leads to a profound dysregulation of gene expression, promoting neuroinflammation, cell death, and impaired neuronal function during sepsis.

The integrity and function of the BBB and neurovascular unit (NVU) are fundamental to brain health, with their disruption implicated in numerous neurological disorders [38]. Key molecular players, including CLDN1, NTF3, RUNX3, and NFKB1Z, exert distinct yet interconnected roles within the cerebral cortex, midbrain, and hippocampus (Fig. 8). CLDN1, a tight junction protein, is minimally expressed under healthy conditions but becomes highly expressed in pathology, driving BBB leakiness and impeding recovery in regions like the cerebral cortex and hippocampus [39]. NTF3, a neurotrophin, supports neuronal survival and plasticity across these brain regions, indirectly fostering a stable NVU [40]. RUNX3, a transcription factor, influences neuronal and glial development, thereby potentially impacting the establishment and maintenance of NVU components [41]. Lastly, Conversely, NFKB1Z, an NF-κB suppressor, emerges as an anti-inflammatory mediator [42] within the NVU, promoting cytokine production and glial reactivity, particularly in response to BBB disruption, with implications for neuroinflammation and neuronal injury in all three regions. Together, these genes represent critical nodes in the regulation of BBB integrity, neuroinflammatory responses, and neurovascular communication, highlighting potential therapeutic targets for mitigating brain dysfunction in sepsis-associated encephalopathy.

Our current study primarily focused on the pathophysiology of endothelial *Ezh2* deficiency during active polymicrobial sepsis. However, many experiments evaluating the sepsis-independent roles of EZH2 were not performed. Specifically, we did not evaluate whether *Ezh2* knockout in the absence of sepsis can independently induce BBB hyperpermeability (measured by albumin extravasation), neuronal loss, microglial and astrocytic activation, or oligodendrocyte proliferation. Therefore, it remains possible that some of the observed phenotypes are sepsis-independent, which warrants thorough investigation in future homeostatic studies. A technical limitation of our study is the assessment of cerebral edema. While we recorded wet whole-brain weights following the challenge, we did not measure dry brain weights to calculate the precise wet-to-dry weight ratio. Future studies will utilize the standard drying method to more accurately quantify brain water percentage.

## Discussion

SAE represents a severe neurological complication of sepsis, characterized by cognitive dysfunction and increased mortality [43]. While the role of the epigenetic regulator *Ezh2* in inflammation has been extensively studied, its specific functions within vascular endothelial cells and their contribution to systemic inflammatory responses and organ dysfunction, particularly in the brain during sepsis, have remained incompletely understood. Here, we present the first comprehensive report demonstrating a critical role for endothelial-specific *Ezh2* in maintaining vascular integrity and mitigating neuroinflammatory pathology during sepsis.

*Ezh2*, as a core component of Polycomb Repressive Complex 2 (PRC2), functions as a histone methyltransferase, primarily catalyzing the trimethylation of histone H3 lysine 27 (H3K27me3) [44]. This epigenetic modification is generally associated with transcriptional repression [45, 46]. Utilizing an astrocyte-specific *Ezh2* cKO mouse model, a previous work examined the effects of *Ezh2* deletion on astrocyte morphogenesis, BBB integrity and neurodevelopment. The loss of *Ezh2* resulted in increased GFAP expression, altered astrocyte morphology and reduced coverage of astrocytic end feet on blood vessels, thereby compromising BBB integrity [30].

Therefore, it is plausible that in endothelial cells, *Ezh2* represses the expression of genes that actively destabilize tight junctions or inhibit angiogenesis, or conversely, it may indirectly promote the expression of genes essential for BBB integrity by repressing their repressors. Further studies exploring the direct and indirect transcriptional targets of *Ezh2* in brain endothelial cells are warranted to fully elucidate these mechanisms.

Interestingly, we observed that constitutive endothelial deletion of *Ezh2* using the Tek-Cre driver did not result in the embryonic lethality previously reported in a previous work [29]. We hypothesize that this phenotypic discrepancy may stem from the specific targeting of different exons within the *Ezh2* gene. While some models utilize deletions in early exons that result in a complete null phenotype through nonsense-mediated decay [47, 48], our model may involve the deletion of exons that allow for the expression of a truncated protein or trigger different degrees of genetic compensation by *Ezh1* [49]. Such truncated isoforms might retain sufficient non-canonical scaffolding functions or residual methyltransferase activity to support early vascular development, while still being insufficient to maintain the BBB under the stress of systemic inflammation in adulthood. This highlights the importance of the specific genomic targeting strategy when evaluating the biological roles of epigenetic regulators like *Ezh2*.

The integrity of the BBB is paramount for maintaining CNS homeostasis, regulating the brain microenvironment, and protecting against neurotoxic substances and systemic inflammation [50, 51]. Our study elucidates a novel and critical role for endothelial *Ezh2* in sustaining BBB integrity and mitigating brain injury during SAE. Our findings demonstrate that endothelial-specific *Ezh2* deletion leads to structural and functional BBB impairment, which significantly exacerbates neuroinflammation and neuronal death in a clinically relevant sepsis model. For the wet weight of whole brain, however, apparent reduction were not observed in both cKO model and iKO model (Figure S2b, 2c). Since the *Ezh2* deletion in iKO mice was induced at adulthood (8–12 weeks), the brain had already completed its primary development. The brief interval between induction and tissue harvest was insufficient to cause gross morphological changes in brain volume. For cKO model, potential imaging artifact or specific regional effect might be one of the reasons. *Ezh2* is often involved in specific cell lineages, like the lining of blood vessels. If *Ezh2* deletion only affects endothelial cells, the total brain volume and weight might remain unchanged. Moreover, other Polycomb Repressive Complex 2 (PRC2) components, such as *Ezh1*, may provide a compensatory buffer in the adult brain that prevents immediate tissue loss following the deletion of *Ezh2*.

Our initial characterization of endothelial *Ezh2* cKO mice revealed profound alterations in BBB components. Specifically, we observed a significant reduction in Claudin-5 expression in endothelial cells, a key component of tight junctions. Tight junctions are crucial for maintaining the restrictive permeability of the BBB, and their disruption is a hallmark of various neurological disorders [51–55]. The quantitative analysis of cerebral vasculature further showed exhibited a moderate increase in 3D vessel volume, number of segments, total vessel length, and reduction in mean radius in *Ezh2* cKO mice. This suggests that *Ezh2* is not merely involved in modulating tight junction protein expression but also plays a broader role in regulating cerebral vascular development and maintenance.

The compromised BBB observed in *Ezh2* cKO mice provided a strong rationale for investigating their susceptibility to systemic inflammatory insults. Sepsis, a life-threatening organ dysfunction caused by a dysregulated host response to infection, frequently leads to SAE, characterized by diffuse brain dysfunction, neuroinflammation, and neuronal apoptosis [56, 57]. Our in vivo sepsis model, induced by cecal ligation and puncture (CLP), demonstrated a dramatic decrease in survival rates and a significant increase in neuronal and parenchymal cell death in *Ezh2* iKO-CLP mice compared to WT-CLP controls. This highlights the vital protective role of endothelial *Ezh2* in maintaining brain resilience during severe systemic inflammation. The higher M-CASS scores in *Ezh2*-deficient mice (Fig. 4i) suggest that the absence of EZH2-mediated H3K27me3 (histone H3 lysine 27 trimethylation) may lead to the “leaky” transcription of pro-inflammatory cytokines, potentially accelerating the transition from a localized infection to a lethal systemic cytokine storm.

The lack of macroscopic Evans Blue leakage, despite significant micro-vascular breaches and glial activation, underscores a nuanced role for *Ezh2*. It suggests that *Ezh2* loss doesn’t cause an immediate, open-hole collapse, but rather lowers the barrier height, allowing inflammatory mediators and smaller macromolecules to infiltrate the CNS and trigger the subsequent encephalopathy phenotype. This independent homeostasis role of the endothelium warrants further investigation into *Ezh2*’s regulation of transcellular vs. paracellular transport pathways. The exacerbation of brain injury in *Ezh2* iKO mice underscores the direct link between endothelial *Ezh2*, BBB integrity, and neuroprotection during sepsis. This is consistent with accumulating evidence suggesting that BBB dysfunction is a critical initiating event in SAE, allowing the influx of inflammatory mediators and pathogens into the CNS [58]. Our findings suggest that endothelial *Ezh2* acts as a crucial guardian against sepsis-induced neurological complications by preserving BBB function. Utilizing the well-established CLP model to induce sepsis, we uncovered a dramatic increase in mortality rates in both *Ezh2* cKO and *Ezh2* iKO mice compared to wild-type controls. This heightened susceptibility to sepsis was accompanied by a significant promotion of inflammatory responses and extensive neuronal death across multiple brain regions, including the cortex, hippocampus, and midbrain. Specifically, toluidine blue staining revealed a marked increase in the percentage of apoptotic and degenerating cells in the cortex, parenchyma, midbrain, and hippocampus of *Ezh2*-deficient septic mice. These findings underscore the critical role of endothelial *Ezh2* in protecting the brain from sepsis-induced injury.

By decoupling survival from neurofunctional performance, we showed that the loss of endothelial *Ezh2* directly sensitizes the brain to inflammatory insults. This suggests that *Ezh2*-mediated epigenetic regulation is a front-line defense at the vascular interface. When this defense is compromised, the brain parenchyma is exposed to excessive inflammatory infiltration and cytokine storms, leading to functional behavioral deficits that precede or occur independently of fatal systemic collapse.

Moreover, our study revealed distinct alterations in glial cell reactivity in *Ezh2*-deficient septic mice. While microglial (IBA1-positive) and astrocyte (GFAP-positive) reactivity were markedly activated in the cortex, their responses were notably suppressed in the hippocampus of *Ezh2*-deficient mice compared to wild-type controls. This region-specific modulation of glial responses highlights the complex interplay between endothelial *Ezh2* and neuroimmune interactions during sepsis [59]. Beyond direct neuronal death, our results also revealed significantly heightened glial cell activation, specifically astrogliosis and microglial activation, in the brains of *Ezh2* iKO-CLP mice. These can reflect the transition of the nervous system from normal physiological states to various pathological states, including nerve damage [60], neurodegenerative diseases [61], tumors [62], and neuroinflammation [63]. These cells not only play a key role in maintaining the normal function of the CNS, but also play an important role in the occurrence and development of diseases. In our study, we observed a significant increase in Olig2-positive cells in the cortex of *Ezh2* iKO-CLP mice compared to their wild-type counterparts. This finding is consistent with literature describing reactive oligodendrogenesis or OPC proliferation in response to acute cortical injury and BBB disruption [64–66]. Previous work have demonstrated that Olig2 can be upregulated in reactive glia following CNS insult, often driven by astrocyte-derived mitogens like Sonic Hedgehog (SHH) [67]. We speculate that the exacerbated BBB leakage and subsequent increase in cortical astrogliosis in *Ezh2*-deficient mice creates a robust pro-proliferative environment for the oligodendrocyte lineage. The lack of a similar response in the hippocampus may reflect regional differences in the neuroinflammatory niche or a higher vulnerability to sepsis-induced cell loss in hippocampal circuits, as previously noted in models of sepsis-associated encephalopathy [68].

Astrocytes and microglia are integral components of the neurovascular unit, and their reactive states contribute to both protective and detrimental effects on the CNS [69, 70]. In the context of sepsis, excessive and prolonged activation of these glial cells can perpetuate neuroinflammation and contribute to neuronal damage [71]. The increased activation observed in our model strongly suggests that the absence of endothelial *Ezh2* amplifies the neuroinflammatory cascade in response to systemic infection. This could be due to increased leakage of pro-inflammatory cytokines and immune cells across the impaired BBB, directly stimulating glial responses. Alternatively, endothelial *Ezh2* might directly or indirectly regulate the secretion of factors that modulate glial activation, a hypothesis that warrants further investigation. The regional differences in oligodendrocyte response (increased Olig2 positive cells in cortex but not hippocampus) could reflect distinct vulnerabilities or regenerative capacities of different brain regions to *Ezh2* deficiency and sepsis.

To elucidate the molecular underpinnings of these observations, we performed comprehensive transcriptomic analyses of the cortex, hippocampus, and midbrain. These analyses revealed distinct alterations in gene expression profiles associated with neuronal, microglial, and astrocytic responses in *Ezh2*-deficient septic mice. Upregulated pathways consistently pointed towards inflammatory responses, immune cell activation, and cell death pathways, while downregulated pathways often included those related to neuronal function and metabolic processes. The specific changes in gene networks and upstream regulators further underscore the pivotal function of *Ezh2* in preserving vascular integrity and suppressing the onset, progression, and severity of SAE through the regulation of inflammatory pathways and neuroimmune interactions [72]. This hypothesis underscores the importance of epigenetic regulation in vascular and neurological health.

Our comprehensive transcriptomic analysis provided molecular insights into the observed phenotypic changes. The RNA-seq data from *Ezh2* iKO-CLP brains revealed significant upregulation of genes associated with immune and inflammatory responses, alongside downregulation of genes related to synaptic function and neuronal development. Pathway enrichment analysis identified key inflammatory signaling pathways, such as TNF signaling and NF-κB signaling, as significantly activated. This suggests that endothelial *Ezh2* normally exerts a repressive effect on these pro-inflammatory pathways. Loss of endothelial *Ezh2* could lead to uncontrolled activation of these pathways, driving the neuroinflammatory environment and subsequent neuronal damage. This is consistent with previous studies demonstrating *Ezh2*’s role in regulating immune responses in T cells and macrophages [73, 74]. Our data suggest that this regulatory role extends to the vascular endothelium within the neurovascular unit, influencing the brain’s response to systemic inflammation. The suppression of synaptic and neuronal developmental pathways further indicates a broader impact on neuronal function and recovery, which could underlie the long-term cognitive deficits often observed in SAE survivors [75].

While our study provides robust evidence for the critical role of endothelial *Ezh2*, certain limitations should be acknowledged. Firstly, while Tek-Cre and Tek-CreERT2 are commonly used for endothelial-specific deletion, complete specificity cannot be entirely guaranteed, as some Tek expression might exist in other hematopoietic or mesenchymal lineages [76]. However, the consistent and profound endothelial-specific *Ezh2* reduction observed in our immunofluorescence studies largely mitigates this concern. Secondly, the CLP model, while clinically relevant, represents a severe form of sepsis [77]. Future studies could explore the role of endothelial *Ezh2* in more moderate models of inflammation or in the context of chronic neuroinflammatory conditions. Lastly, while our transcriptomic data highlight dysregulated pathways, direct evidence of *Ezh2*’s downstream targets in brain endothelial cells relevant to BBB integrity and neuroinflammation is still needed. Chromatin immunoprecipitation sequencing (ChIP-seq) in isolated brain endothelial cells would be invaluable to identify direct EZH2 binding sites and their associated gene targets.

The choroid plexus serves as a specialized neuro-immune interface. In our study, the loss of endothelial *Ezh2* significantly promoted the recruitment of Ly6G^+^ neutrophils and CD3^+^ T cells (CD4^+^ and CD8^+^ subtypes) to the choroid plexus following sepsis. This is particularly noteworthy as the choroid plexus is a primary site for leukocyte trafficking into the CSF. The enrichment of neutrophils (Ly6G^+^) suggests an intensified acute innate immune response, while the increased T cell (CD3^+^) presence points to a breakdown of the immune-privileged status of the CSF compartment. We speculate that *Ezh2* deficiency might upregulate adhesion molecules (such as ICAM-1 or VCAM-1) or chemokines in the choroid plexus capillaries, actively ‘pulling’ these peripheral effectors into the CNS. This influx likely amplifies the secondary inflammatory cascade in the brain parenchyma, explaining the exacerbated SAE clinical scores and glial reactivity observed in *Ezh2* iKO mice.

Our study highlights endothelial *Ezh2* as a novel and potential therapeutic target for SAE. The ability of endothelial *Ezh2* to modulate both vascular integrity and neuroinflammatory dynamics that targeting this epigenetic regulator could offer a unique approach to mitigating sepsis-induced complications. While our models demonstrate a clear role for endothelial *Ezh2*, future studies could further delineate the specific downstream targets of *Ezh2* in endothelial cells that contribute to BBB integrity and neuroprotection during sepsis. Additionally, investigating the precise mechanisms by which endothelial *Ezh2* influences regional differences in glial responses would provide valuable insights.

## Conclusions

In summary, our study identifies endothelial *Ezh2* as a key epigenetic regulator that maintains BBB integrity and limits neuroinflammatory activation during sepsis. Loss of endothelial *Ezh2* establishes a vulnerable neurovascular state characterized by impaired tight junction expression, altered vascular morphology, and reduced H3K27me3-mediated repression, which becomes rapidly destabilized under systemic inflammatory challenge. This vulnerability promotes extensive leukocyte infiltration, region-specific microglial and astrocytic reactivity, neuronal apoptosis, and behavioral dysfunction, culminating in aggravated SAE severity and reduced survival. Transcriptomic profiling across multiple brain regions reveals coordinated dysregulation of inflammatory, metabolic, and synaptic pathways, providing molecular insight into how endothelial epigenetic dysfunction amplifies peripheral-CNS immune communication. These findings underscore the critical role of the vascular endothelium in shaping CNS inflammatory responses and identify endothelial *Ezh2* as a neurovascular checkpoint that restrains pathological immune entry into the brain. Targeting endothelial epigenetic mechanisms may offer a novel avenue for mitigating neuroinflammation and reducing CNS complications associated with systemic inflammation.

## Supplementary Information

Supplementary Material 1.

## Data Availability

No datasets were generated or analysed during the current study.

## Abbreviations

ANOVA: Analysis of Variance
BBB: Blood-Brain Barrier
CD31: Platelet Endothelial Cell Adhesion Molecule-1
CLP: Cecal Ligation and Puncture
cKO: Conditional Knockout
CreERT2: Tamoxifen-Inducible Cre Recombinase
DAPI: 4’,6-Diamidino-2-Phenylindole
DEGs: Differentially Expressed Genes
dpi: Days Post-Injury
Ezh2: Enhancer of Zeste Homolog 2
FACS: Fluorescence-Activated Cell Sorting
FDR: False Discovery Rate
GFAP: Glial Fibrillary Acidic Protein
G/R: Green/Red (reporter mice)
hpi: Hours Post-Injury
H&E: Hematoxylin and Eosin
H3K27me3: Histone H3 Lysine 27 Trimethylation
IBA1: Ionized Calcium-Binding Adapter Molecule 1
iKO: Inducible Knockout
KEGG: Kyoto Encyclopedia of Genes and Genomes
O.C.T.: Optimal Cutting Temperature
Olig2: Oligodendrocyte Transcription Factor 2
PBS: Phosphate-Buffered Saline
PFA: Paraformaldehyde
PI: Propidium Iodide
PPI: Protein-Protein Interaction
PRC2: Polycomb Repressive Complex 2
RNA-seq: RNA Sequencing
SAE: Sepsis-Associated Encephalopathy
WT: Wild-Type
