# Supplementary material for: Endothelial-specific Ezh2 deficiency exacerbates blood-brain barrier dysfunction and neuroinflammation in sepsis-associated encephalopathy
Source: J Neuroinflammation. 2026 Apr 7;23:167. doi: 10.1186/s12974-026-03798-z (PMC13200478; doi:10.1186/s12974-026-03798-z)
Supplement: Supplementary file 1 — Supplementary Material 1. [file 12974_2026_3798_MOESM1_ESM.docx]

Figure S1


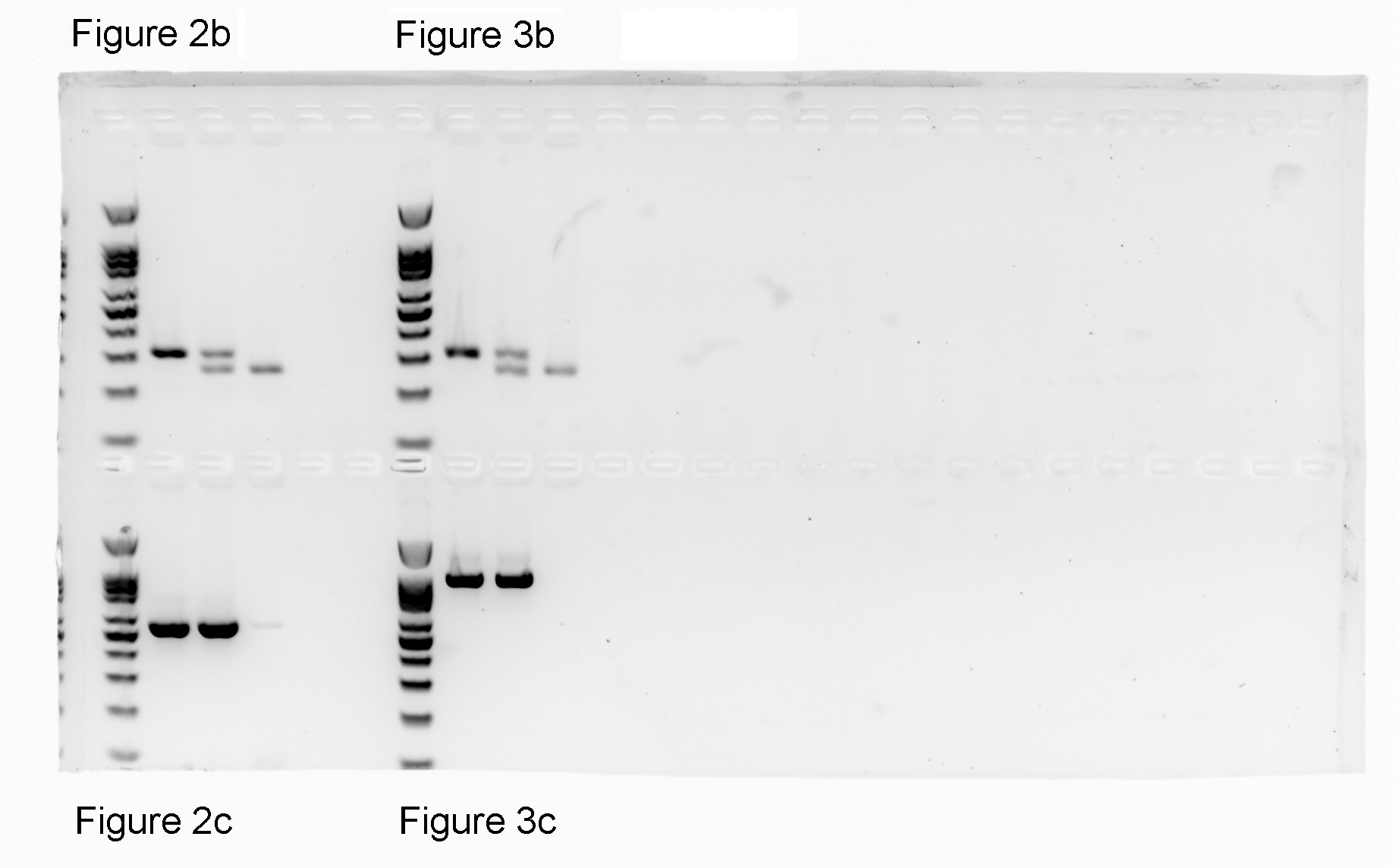


**Figure S1 | Original uncropped agarose gel images.** This figure displays the full-length, unedited agarose gel electrophoresis images used to generate the cropped panels in the main manuscript. These images confirm the specificity of the primers used and the absence of non-specific amplification or contamination.

Figure S2


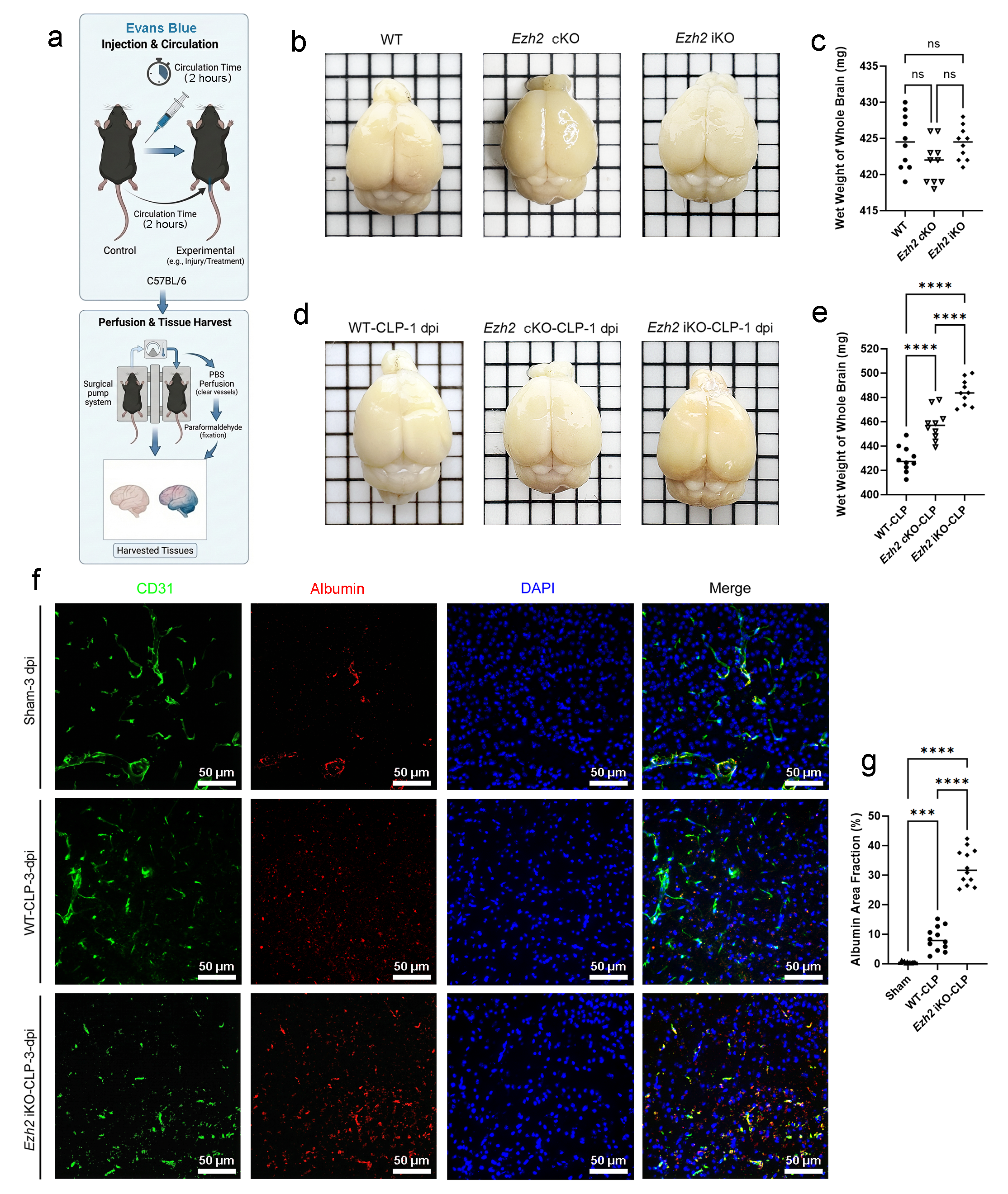


**Figure S2 | Loss of *Ezh2* exacerbates brain edema and BBB leakage following sepsis.** (a) Schematic workflow of the experimental procedure, highlighting systemic Evans Blue injection, circulation time (2 hours), and subsequent transcardial perfusion for tissue harvesting in C57BL/6 mice. (b) Representative gross anatomical images of harvested whole brains from WT, *Ezh2* cKO, and *Ezh2* iKO mice under baseline physiological conditions, confirming the absence of gross, macroscopically visible Evans Blue dye extravasation into the brain parenchyma. (c) Quantification showing no significant difference (ns) in baseline brain wet weight between groups. (d) Representative gross anatomical images of brains from septic WT, *Ezh2* cKO, and *Ezh2* iKO mice at 1 day post-infection (dpi) following Cecal Ligation and Puncture (CLP), demonstrating a lack of overt structural collapse or non-specific macroscopic Evans Blue dye leakage. (e) Quantification of whole brain wet weight, indicating significantly increased edema in *Ezh2* iKO-CLP mice compared to WT-CLP and *Ezh2* cKO-CLP groups. (f) Representative immunofluorescence images of midbrain sections at 3 dpi stained for CD31 (endothelial cells, green), albumin (extravasation marker, red), and DAPI (nuclei, blue). Scale bars = 50 μm. (g) Quantitative analysis of the albumin area fraction (%). Data demonstrate that *Ezh2* deficiency significantly increases BBB permeability following sepsis induction compared to the WT-CLP group. Data are presented as individual values with the mean (n = 12 per group). Statistical significance was determined by one-way ANOVA with Tukey’s post hoc test. ****p* < 0.001, *****p* < 0.0001, ns = not significant.

Figure S3


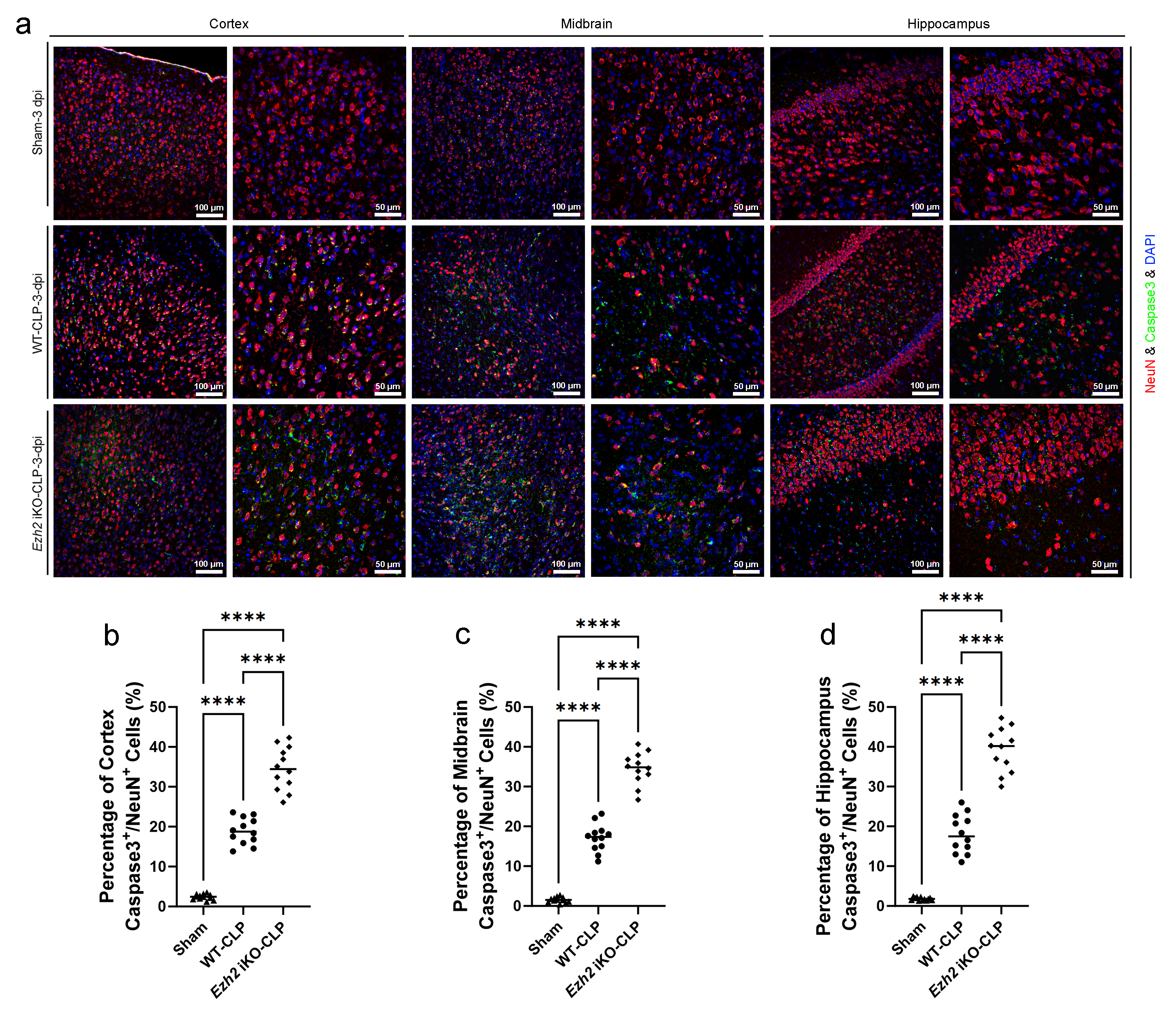


**Figure S3 | Ezh2 deficiency exacerbates neuronal apoptosis in multiple brain regions following sepsis.** (a) Representative immunofluorescence images of the cortex, midbrain, and hippocampus at 3 days post-injury (dpi). Brain sections were stained for NeuN (red; mature neurons), Caspase-3 (green; apoptotic marker), and DAPI (blue; nuclei). Top row: Sham-operated group; Middle row: Wild-type (WT) mice subjected to CLP; Bottom row: *Ezh2* inducible knockout (iKO) mice subjected to CLP. Scale bars: 100 μm (low magnification) and 50 μm (high magnification). (b-d) Quantitative analysis of the percentage of apoptotic neurons (Caspase-3^+^/NeuN^+^ cells) in the (b) cortex, (c) midbrain, and (d) hippocampus across the three experimental groups. Data are presented as individual values with the mean (n = 12 per group). Statistical significance was determined by one-way ANOVA followed by Tukey’s post hoc test. *****p* < 0.0001.

Figure S4


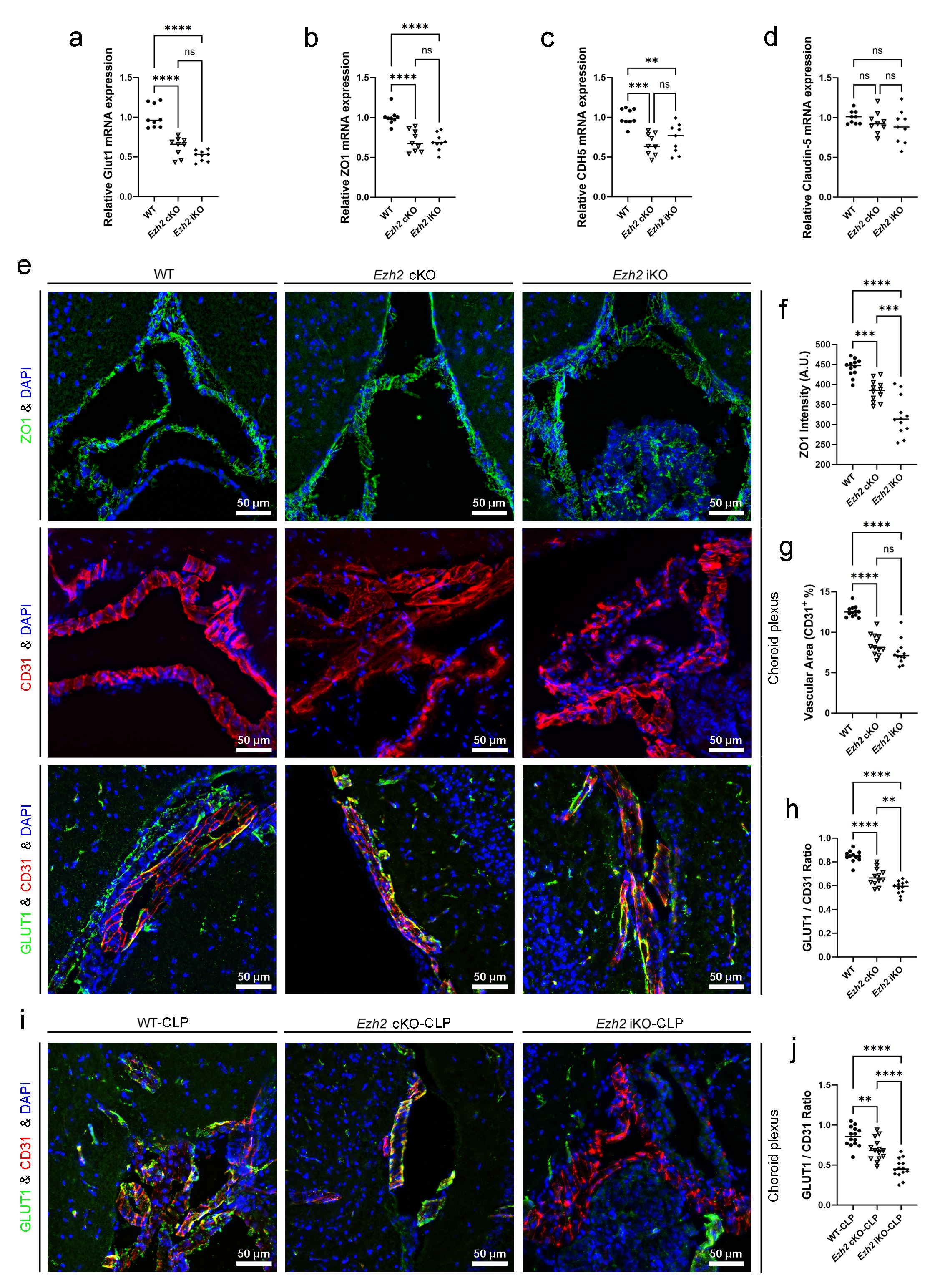


**Figure S4 | EZH2 is essential for the maintenance of Choroid Plexus barrier integrity and vascular identity.** (a-d) Quantitative RT-PCR analysis of mRNA expression levels for key barrier and transport markers in the forebrain cortex of WT, *Ezh2* cKO, and *Ezh2* iKO mice. Markers include (a) *Glut1* (glucose transporter), (b) *Zo1* (tight junction protein), (c) *CDH5* (Cadherin 5), and (d) *Cldn5* (note: panel d shows no significant change in *Claudin-5* mRNA across groups). Data are presented as individual values with the mean; n=9 per group. *****p* < 0.0001, ****p* < 0.001$, ***p* < 0.01, ns = non-significant by one-way ANOVA with Tukey’s post-hoc test. (e) Representative immunofluorescence images of the choroid plexus sections stained for ZO1 (green, top row), CD31 (red, middle row), and merged GLUT1/CD31 (bottom row). Nuclei are counterstained with DAPI (blue). Scale bars = 50 μm. (f-h) Quantification of protein expression and vascular morphology based on the images in (e). (f) Mean fluorescence intensity of ZO1, showing a significant reduction in tight junction protein expression in *Ezh2* iKO tissues. (g) Percentage of Vascular Area (CD31^+^  area), indicating a reduction in vascular density in both cKO and iKO models. (h) Ratio of GLUT1 to CD31 fluorescence, highlighting a loss of specialized barrier transporter expression relative to the vascular endothelial surface area. (i-j) Analysis of barrier integrity under inflammatory stress (CLP: Cecal Ligation and Puncture model). (i) Immunofluorescence images of GLUT1 (green) and CD31 (red) in WT, cKO, and iKO mice following CLP-induced systemic inflammation. (j) Quantification of the GLUT1/CD31 ratio under CLP conditions, demonstrating that EZH2 deficiency exacerbates the loss of endothelial GLUT1 expression during inflammatory challenge.

Figure S5


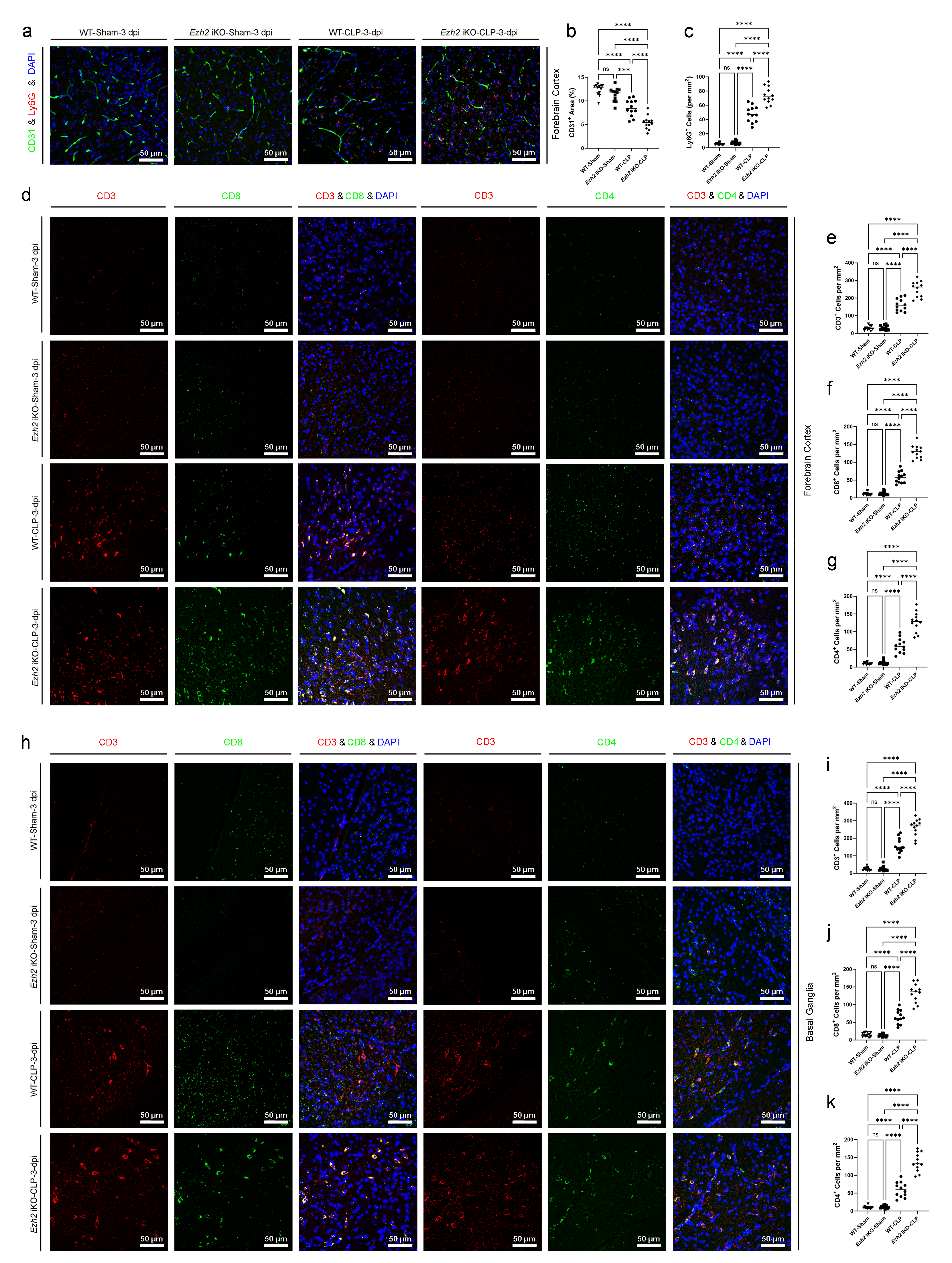


**Figure S5 | Ezh2 deficiency in endothelial cells exacerbates sepsis-induced neuroinflammation and T-cell infiltration.** (a) Representative immunofluorescence images of the forebrain cortex at 3 days post-infection (dpi) showing CD31 (green, endothelial marker), Ly6G (red, neutrophil marker), and DAPI (blue, nuclei) in WT-Sham, *Ezh2* iKO-Sham, WT-CLP, and *Ezh2* iKO-CLP mice. Scale bars = 50 μm. (b-c) Quantification of (b) CD31^+^ vessel area (%) and (c) Ly6G^+^ neutrophil infiltration per mm^2^ in the forebrain cortex. Sepsis significantly reduces vascular density and increases neutrophil recruitment, effects which are exacerbated by the loss of *Ezh2*. (d) Representative confocal images of the forebrain cortex stained for CD3 (red), CD8 (green), CD4 (green), and DAPI (blue). The panels illustrate the recruitment of T-cell subsets (CD3^+^, CD8^+^, and CD4^+^) into the brain parenchyma following CLP. Scale bars = 50 μm. (e-g) Quantitative analysis of (e) CD3^+^, (f) CD8^+^, and (g) CD4^+^ T cells per mm^2^ in the forebrain cortex across the four experimental groups. (h) Representative immunofluorescence images of the Basal Ganglia at 3 dpi, stained for CD3 (red), CD8 (green), CD4 (green), and DAPI (blue), showing regional neuroinflammatory response. Scale bars = 50 μm. (j-k) Quantification of (i) CD3^+^, (j) CD8^+^, and (k) CD4^+^ T cells per mm^2^ in the basal ganglia. Statistical Analysis: Data are presented as individual values with the mean (n = 12 mice per group). Statistical significance was determined by Two-way ANOVA followed by Tukey’s post-hoc test. ns, non-significant; **p < 0.05*; ***p* < 0.01;****P <* 0.001; *****p <* 0.0001.

Figure S6


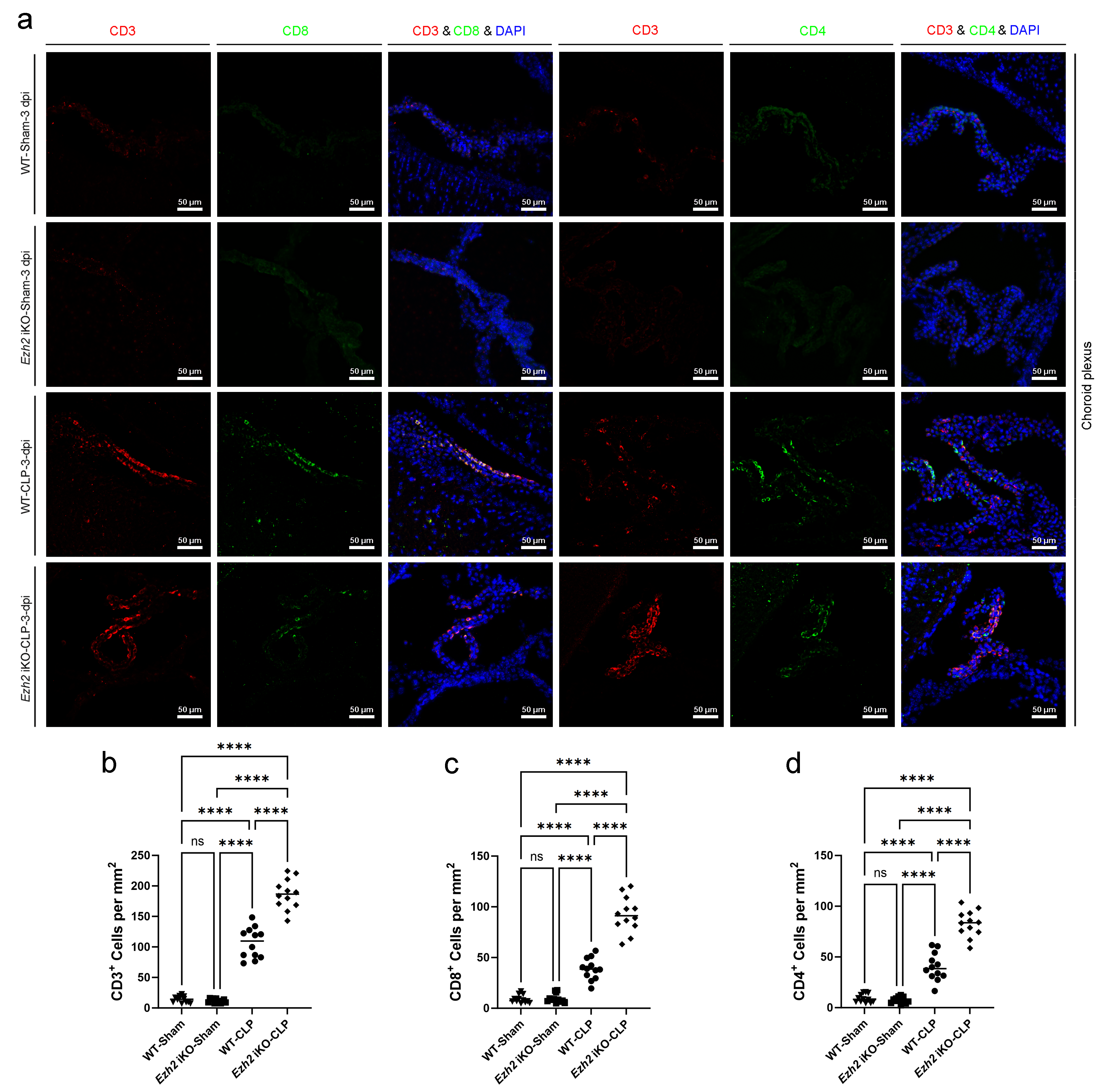


**Figure S6 | Ezh2 deficiency exacerbates T cell infiltration into the choroid plexus during sepsis.** (a) Representative immunofluorescence images of the choroid plexus at 3 days post-infection (dpi). Brain sections from WT and *Ezh2* iKO mice were stained for CD3 (red), CD8 (green), CD4 (green), and DAPI (blue) following Sham or CLP surgery. Scale bars = 50 μm. The images demonstrate a marked increase in the presence of CD3^+^, CD8^+^, and CD4^+^ T cells within the choroid plexus of CLP-challenged mice, which is further amplified by the loss of *Ezh2*. (b-d) Quantitative analysis of infiltrating T cell subsets per mm^2^ in the choroid plexus: (b) Total CD3^+^ T cells. (c) CD8^+^ cytotoxic T cells. (d) CD4^+^ helper T cells. Data are presented as individual values with the mean (n = 12 mice per group). Statistical significance was determined using Two-way ANOVA followed by Tukey’s post hoc test. ns, non-significant; *****p* < 0.0001.

Table S1. Mouse Clinical Assessment Score for Sepsis (M-CASS)

| Score | 0 | 1 | 2 | 3 |
| --- | --- | --- | --- | --- |
| Fur Aspect | Normal fur | Slightly ruffled fur | Ruffled fur | Ruffled fur and piloerection |
| Activity | Normal | Reduced | Only when provoked | Little or none with provocation |
| Posture | Normal | Hunched, moving freely | Hunched, strained or stiff movement | Hunched, little or no movement |
| Behavior | Normal | Slow | Abnormal when disturbed or provoked | Abnormal, no relocation |
| Chest Movements | Normal | Mild dyspnea | Moderate dyspnea | Severe dyspnea |
| Eyelids | Normal, open | Opened when disturbed | Partially closed, even when disturbed | Mostly or completely closed, even when provoked |
